# Phage induction of *Staphylococcus aureus* pathogenicity islands promotes the CRISPR-Cas adaptive immune response

**DOI:** 10.1016/j.celrep.2025.116776

**Published:** 2025-12-30

**Authors:** Dalton V. Banh, Gregory W. Goldberg, Luciano A. Marraffini

**Affiliations:** 1 Laboratory of Bacteriology, The Rockefeller University, New York, NY, USA; 2 Weill Cornell/Rockefeller/Sloan Kettering Tri-Institutional MD-PhD Program, New York, NY, USA; 3 Howard Hughes Medical Institute, The Rockefeller University, New York, NY, USA; 4 Present address: Institute for Systems Genetics, NYU Langone Health, New York, NY, USA; 5 Senior author; 6 Lead contact

## Abstract

*Staphylococcus aureus* pathogenicity islands (SaPIs) are mobile genetic elements carrying virulence genes that spread upon infection by helper phages that induce their transfer. Staphylococci also carry type II and III CRISPR-Cas systems that mount an adaptive immune response against phages through the acquisition of spacer sequences from viral genomes, directing Cas nucleases to their targets. Whether and how SaPIs and CRISPR interact with each other during helper phage infection is not known. Here we report that, as a result of the packaging of incomplete helper phage genomes into SaPI particles, defective viral DNA delivered into new hosts stimulates spacer acquisition in both CRISPR types. Once immunized, staphylococci target the helper phage and prevent SaPI mobilization. Our work reveals an unexpected synergy between CRISPR-Cas systems and SaPIs that enhances antiphage immunity and could favor the retention of beneficial elements within the population.

## In brief

Banh et al. show that staphylococci carrying CRISPR-Cas systems and SaPIs display enhanced rates of CRISPR spacer acquisition from phages. This requires delivery of incomplete, non-replicating phage genomes by SaPIs, which, as opposed to fully infective phages that rapidly kill the cell, allows CRISPR immunization of the host before its lysis.

## Graphical Abstract

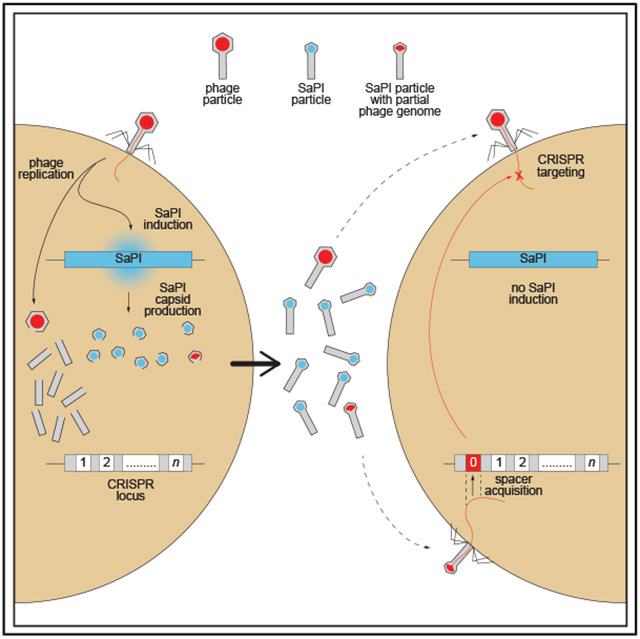

## INTRODUCTION

Bacteria have evolved an immense arsenal of diverse immune strategies to survive an ongoing arms race against their viruses, the bacteriophages (phages). Present in ~40% of cultivated bacteria,^1^ clustered regularly interspaced short palindromic repeat (CRISPR) loci and CRISPR-associated (*cas*) genes comprise adaptive immune systems that act against invading genetic elements, namely phages^2^ and plasmids.^3^ CRISPR loci consist of an array of short (~40 bp) repetitive sequences separated by equally short spacer sequences of phage and plasmid origin.^4-6^ There is an enormous diversity of CRISPR-Cas systems, which are classified based on *cas* gene composition into six types and numerous subtypes, with type I, II, and III systems being the most prevalent.^1^ The CRISPR-Cas immune response is thought to occur in two stages: immunization and targeting. Upon infection, a spacer sequence is extracted from the invading phage DNA and inserted between the repeats of the CRISPR array, a process that generates an immunological memory.^2^ In the second stage, repeats and spacers are transcribed and processed to generate mature CRISPR RNAs (crRNAs),^7,8^ which guide Cas effector complexes to recognize and destroy complementary target sequences (known as protospacers) within the genomes of invading phages.^9-11^ This mechanism makes CRISPR-Cas immunity a unique defense system that enables hosts to continually evolve to recognize and neutralize a broad range of old and new viral threats, including phages that rapidly mutate target sequences and avoid recognition.

Phage-inducible chromosomal islands (PICIs) and PICI-like elements (PLEs) comprise a widespread family of satellite-virus elements that require infection with “helper” phages to propagate among Gram-positive and Gram-negative bacteria.^12-14^ The founding members of the PICI family were first discovered in clinical isolates of *Staphylococcus aureus* and named *S. aureus* pathogenicity islands (SaPIs) due to their causative role in toxic shock syndrome as carriers of the superantigen toxin gene.^15^ SaPIs are maintained as integrated elements in the host chromosome due to the transcriptional control of the Stl repressor, which prevents the expression of genes required for SaPI excision and replication.^16^ This repression is subverted upon infection of the host by a helper phage, which expresses a protein that prevents Stl function, leading to the induction and mobilization of SaPI.^17^ In addition to de-repression, helper phages provide the structural components required for packaging of the SaPI genomes into transducing particles that spread these elements among staphylococci.^18,19^ In order to ensure their own spread over that of the helper phage, SaPIs produce unique structural proteins that lead to the formation of a smaller capsid that packages the element’s genome, but cannot accommodate the full length of the larger phage DNA.^20^ This mechanism, known as capsid size redirection,^20^ creates competition between SaPIs and helper phages for the same structural proteins and leads to a reduction in the formation of viral particles, and therefore can be considered a mode of phage defense. Finally, disruption of the host membrane, mediated by the lysins expressed by the helper phage, enables the release of SaPI particles into the environment. These particles are free to infect new hosts and integrate into their chromosomes, endowing them with accessory genes that may provide survival advantages for adaptation^21^ and virulence.^15^ In addition, in a surprising turn of events, it was recently reported that SaPIs often harbor phagedefense systems.^22^

Although PICIs are much more widespread among staphylococci than CRISPR systems, they co-exist in many genomes (Table S1). Given that both interfere with phage infection, we decided to investigate whether these systems interact to provide defense, as well as the effects of this interaction on the development of the CRISPR immune response and on the spread of PICIs. To this end, we studied type II-A and III-A CRISPR-Cas systems usually present in these bacteria, as well as SaPI1. Staphylococcal type II-A CRISPR systems utilize the Cas9 crRNA-guided nuclease to recognize protospacers on the target DNA, which is cleaved when flanked by the proper protospacer-adjacent motif (PAM) 5′-NNGRRT-3′.^23^ Type III-A systems present in staphylococci encode for the Cas10-Csm multi-subunit complex, which uses the crRNA guide to find complementary sequences within viral transcripts.^24^ Target recognition triggers two activities on the Cas10 subunit of this complex: ssDNA degradation^24,25^ and synthesis of cyclic oligoadenylates.^26,27^ Although not sequence-specific,^25^ the nuclease activity is believed to degrade the ssDNA present in the phage transcription bubble,^24^ and is sufficient to provide anti-viral immunity when the target transcript is expressed early in the phage lytic cycle.^28^ On the other hand, cyclic tetra- or hexa-adenylates act as second messengers that bind and activate the type III-A accessory protein Csm6.^26,27^ Csm6 is a non-specific RNase that degrades both host and phage transcripts, leading to the generation of a dormant cell^28,29^ where the virus cannot propagate. This alternative mechanism enables defense when the target RNA is transcribed late in the lytic cycle; i.e., phage replication is well underway, and DNA degradation alone is insufficient to destroy the accumulated viral genomes.^28^

SaPI1 is a prototypical *S. aureus* PICI that is de-repressed by the Φ80α phage^20^ through expression of Sri, a DnaI-binding protein that inhibits staphylococcal replication during the phage lytic cycle.^17^ In addition to this role, Sri binds Stl, inducing a conformational change and reducing the repressor’s affinity to SaPI1 DNA.^30^ Upon de-repression, SaPI1 excises out of the host chromosome and initiates rolling-circle replication, transcription, and expression of gene products required for transduction. Two of these genes encode for CpmA and CpmB, which redirect the assembly of the icosahedral helper phage Φ80α capsid (T = 7 symmetry)^31^ into a smaller structure with T = 4 symmetry.^32^ Remodeled capsids have approximately one-third the volume of the viral capsids and are thus commensurate with the 15.2 kb SaPI1 genome.^20^ In addition, SaPI1 encodes its own terminase small subunit (TerS), which ensures the packaging of SaPI1 DNA into capsids.^33^ However, some Φ80α DNA can still be packaged into modified SaPI1 capsids.^33^ Given the larger size of the Φ80α genome, 43.9 kb,^34^ only a fraction of the viral DNA can be packaged into SaPI1 capsids; therefore, these particles cannot complete an infectious cycle when they inject their DNA into the staphylococcal recipient.

We observed that Φ80α infection of staphylococci harboring both SaPI1 and a type II-A or III-A CRISPR-Cas system leads to an enhancement in the acquisition of new spacers, mediated by SaPI1 particles harboring incomplete viral genomes. Once programmed to target the helper phage, both type II-A and III-A CRISPR-Cas immunity prevent the induction of SaPI1. Although this element can be mobilized by mutant helper phages that evade CRISPR targeting by a particular spacer sequence, these viral escape variants are ultimately eliminated from the population due to the presence of multiple different spacers that can still target individual mutant viruses. In addition, in the case of type III-A systems, the RNase activity of Csm6 triggered by wild-type phages creates inviable hosts for escaper phages, which, upon infection of dormant cells, can induce the excision but not the transfer of SaPI1. These findings expand the role of bacterial CRISPR-Cas systems as barriers against the dissemination of mobile genetic elements beyond phages and plasmids. Importantly, our work reveals an unexpected synergy between CRISPR-Cas systems and SaPIs, in which induction of these elements by helper phages activates adaptive immunity to limit both phage and SaPI spread.

## RESULTS

### SaPI1 induction stimulates the type II-A CRISPR adaptive response against Φ80α helper phage

While it has been reported that staphylococcal genomes harbor type II-A and III-A CRISPR-Cas systems^35^ as well as PICIs,^36^ there has not been a survey to determine how often these elements co-exist in the same strain. Therefore, we used the CRISPRCasdb tool^37^ to find all the staphylococcal strains harboring CRISPR systems, for a total of 111 genomes: 71 containing type II-A loci, 33 III-A, and 7 with both systems (Table S1). We then looked for PICIs in these genomes using SatelliteFinder^38^ and found them in about half of the CRISPR-containing genomes, 30/71 co-existing with type II-A systems, 17/33 with type III-A, and 2/7 with both. To investigate possible interactions between CRISPR and PICIs in staphylococci, we introduced either the type II-A CRISPR-Cas system of *S. aureus* M06/0171^39^ or the type III-A system of *S. epidermidis* RP62A,^40^ as well as SaPI1,^20^ into the *S. aureus* strain TB4, a derivative of the clinical isolate *S. aureus* Newman that was cured of all its prophages.^41^ Cultures were infected with the helper phage Φ80α-vir,^42^ a lytic variant of Φ80α^34^ that we constructed to avoid the emergence of lysogens resistant to superinfection.

**Figure 1.**
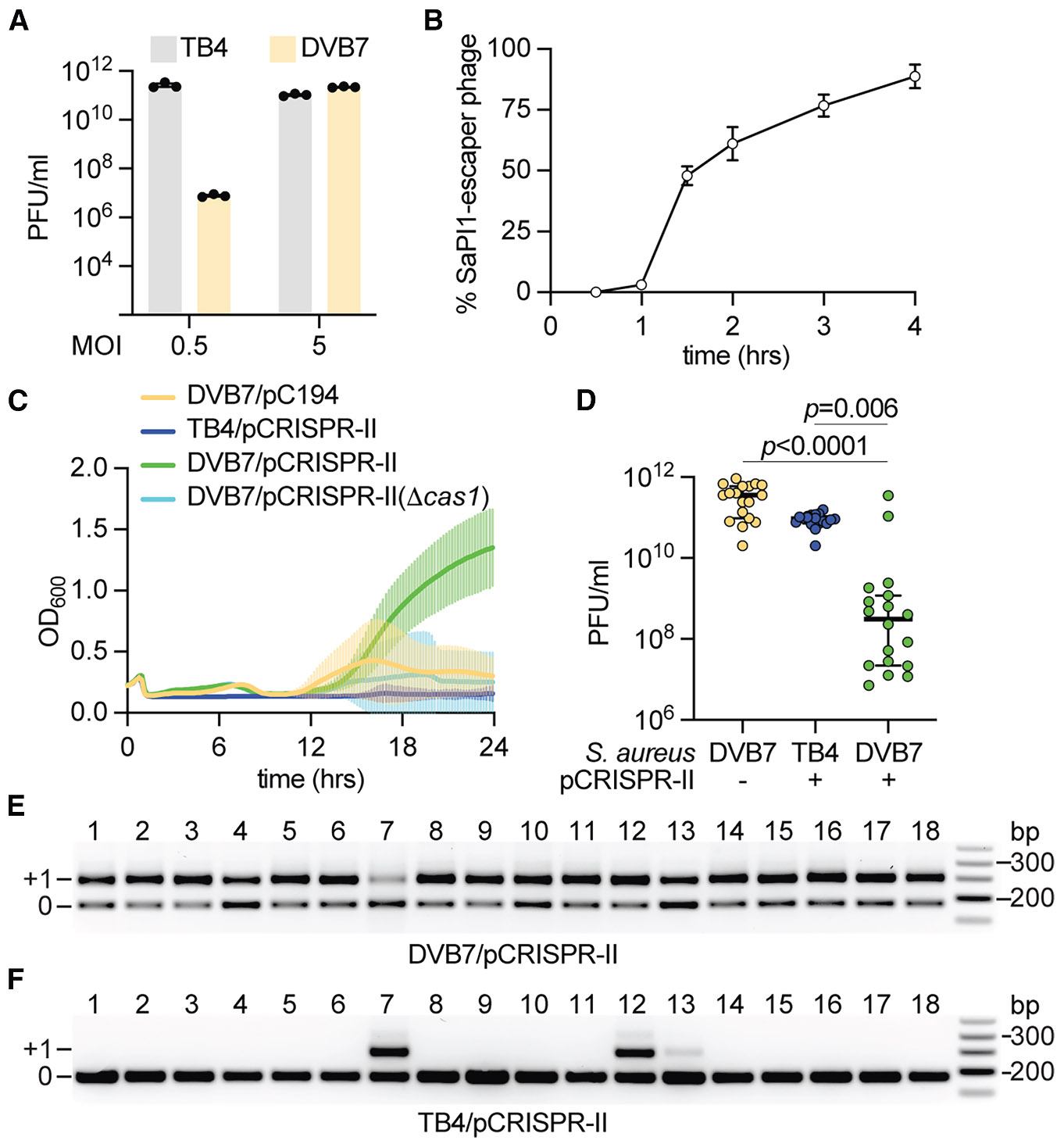
SaPI1 induction stimulates the type II-A CRISPR adaptive response against Φ80α helper phage (A) Enumeration of plaque-forming units (PFU) per milliliter of filtered supernatants taken 4.5 h after infection of *S. aureus* TB4 (SaPI−) or DVB7 (SaPI+) with Φ80α-vir, at a multiplicity of infection (MOI) of 0.5 or 5.0. Mean ± SEM of 3 biological replicates is shown. (B) Fraction (%) of phages that escape SaPI1-mediated capsid size redirection interference, obtained at different times after infection of *S. aureus* DVB7 (SaPI+) with Φ80α-vir, at MOI 10. Mean ± SEM of 3 biological replicates is shown. (C) Growth of different *S. aureus* strains measured by optical density at 600nm (OD_600_) after infection with Φ80α-vir at MOI 10. The mean ± SD for 18 biological replicates is shown. (D) Enumeration of PFUs per milliliter of filtered supernatants taken 24 h after infection of the cultures shown in (C) with a starting phage titer of 1 × 10^9^ PFUs per milliliter. The median ± 95% confidence interval for 18 biological replicates is shown. *p* values were obtained using an unpaired *t* test with Welch’s correction. (E) Detection of spacer acquisition within the type II-A CRISPR locus via PCR. Amplification products obtained using template DNA extracted from 18 infected *S. aureus* DVB7/pCRISPR-II cultures shown in (C) were separated through agarose gel electrophoresis. Products indicated as “+1” show expansion of the CRISPR array with an additional repeat-spacer unit. “0” shows PCR products without such expansion. Molecular weight markers are shown on the right. (F) Same as (E) but using DNA extracted from infected *S. aureus* TB4/pCRISPR-II cultures. See also Figures S1 and S2.

We first characterized the propagation of the helper phage in the cultures of staphylococci harboring SaPI1 only. To this end, we transduced a tetracycline-resistant version of SaPI1 *tst*::*tetM*^15^ into *S. aureus* TB4, generating strain DVB7 (TB4:SaPI1 *tst::tetM*). We then infected cultures of TB4 and DVB7 with Φ80α-vir at different multiplicity of infection (MOI) and measured the optical density (OD_600_) to follow bacterial growth. Consistent with previous results,^43^ at low MOI 0.5, SaPI1 prevented phage propagation (Figure 1A), protecting the uninfected cells from infection and thus enabling the growth of DVB7, but not TB4, cultures (Figure S1A). In contrast, at high MOI 5 and 50, nearly all staphylococci are infected by the helper phage and cultures are rapidly lysed (Figure S1A), producing high Φ80α-vir titers (Figure 1A). Since SaPI1 interferes with helper phage propagation, we wondered whether the phages that accumulate in the culture are mutant viruses that fail to induce SaPI1.^44^ We collected samples at different time points of Φ80α-vir passaged on bacteria harboring SaPI1 and measured their increasing infectivity on DVB7 staphylococci (an experimental approach in evolutionary biology known as a timeshift assay^45^) to trace the rise of SaPI1-escaper viruses during infection (Figure 1B). By the second phage burst (~90 min), 50% of phages released into the supernatant (measured as plaque-forming units, PFUs) were able to evade SaPI1 defense, a fraction that increased to nearly 100% after ~6 generations (4 h). Consistent with previous studies,^44^ isolated escaper phages harbored mutations in the *sri* gene (Figure S1B), which encodes the SaPI1 de-repressor,^17^ and completely evaded SaPI1 defense (Figures S1C and S1D).

We wondered whether a co-occurring CRISPR-Cas system could contain SaPI1 escapers. We started by testing the effect of the type II-A CRISPR-Cas system of *S. aureus* M06/0171,^39^ which we cloned into the staphylococcal plasmid pC194^46^ (Figure S2A). We deleted all pre-existing spacers, generating a minimal CRISPR array containing only a single repeat, to test for the ability of this system to develop adaptive immunity through the acquisition of new spacers. This plasmid, pCRISPR-II, was introduced into TB4 and DVB7 strains, which were infected by Φ80α-vir at MOI 10 (18 independent cultures of each strain were assayed). We chose this relatively high MOI to bypass the SaPI defense and select for cells that survive infection solely due to CRISPR spacer acquisition. We found that DVB7/pCRISPR-II cultures were able to recover from infection using optical density measurements (Figures 1C and S2B). We also measured viral titers and found that 16/18 cultures displayed a drastic reduction (Figure 1D), with the two cultures in which the PFU count remained high, displaying a drop in cell survival at the end of the experiment (cultures #7 and #15, Figure S2B). In contrast, neither DVB7/pC194 (harboring SaPI1 but not the CRISPR locus) nor TB4/pCRISPR-II (lacking SaPI1 but containing the CRISPR system) cultures could overcome phage infection (Figures 1C and S2C), allowing Φ80α-vir proliferation to high titers (Figure 1D). In order to determine if the adaptive response of the type II CRISPR-Cas system was responsible for these phenotypes, we first infected DVB7 cells harboring pCRISPR-II(Δ*cas1*) (Figure S2A) that do not produce the Cas1 integrase required to incorporate new spacers into the CRISPR locus,^47-49^ and found that this culture was not able to regrow (Figure 1C). Similar results were obtained after plating aliquots of the different infected cultures for the enumeration of CFU/mL (Figure S2D). We also used PCR to assay for the expansion of the CRISPR array (Figure S2A) in each of the 18 DVB7/pCRISPR-II cultures. We found that all of them acquired new spacers (Figure 1E) with sequences that matched the Φ80α-vir genome (Figure S2E). On the other hand, in the absence of SaPI1 induction, only 3/18 TB4/pCRISPR-II cultures acquired spacers, #7, 12, and 13 (Figure 1F), which enabled limited regrowth (Figure S2C). This relatively low spacer acquisition level is common for type II-A systems.^47,50^ Altogether, these results show that the staphylococcal type II-A CRISPR-Cas system can mount an adaptive immune response to curb the rise of mutant helper phages that evade SaPI1 interference.

### SaPI1-modified particles carrying partial helper phage genomes enhance spacer acquisition

**Figure 2.**
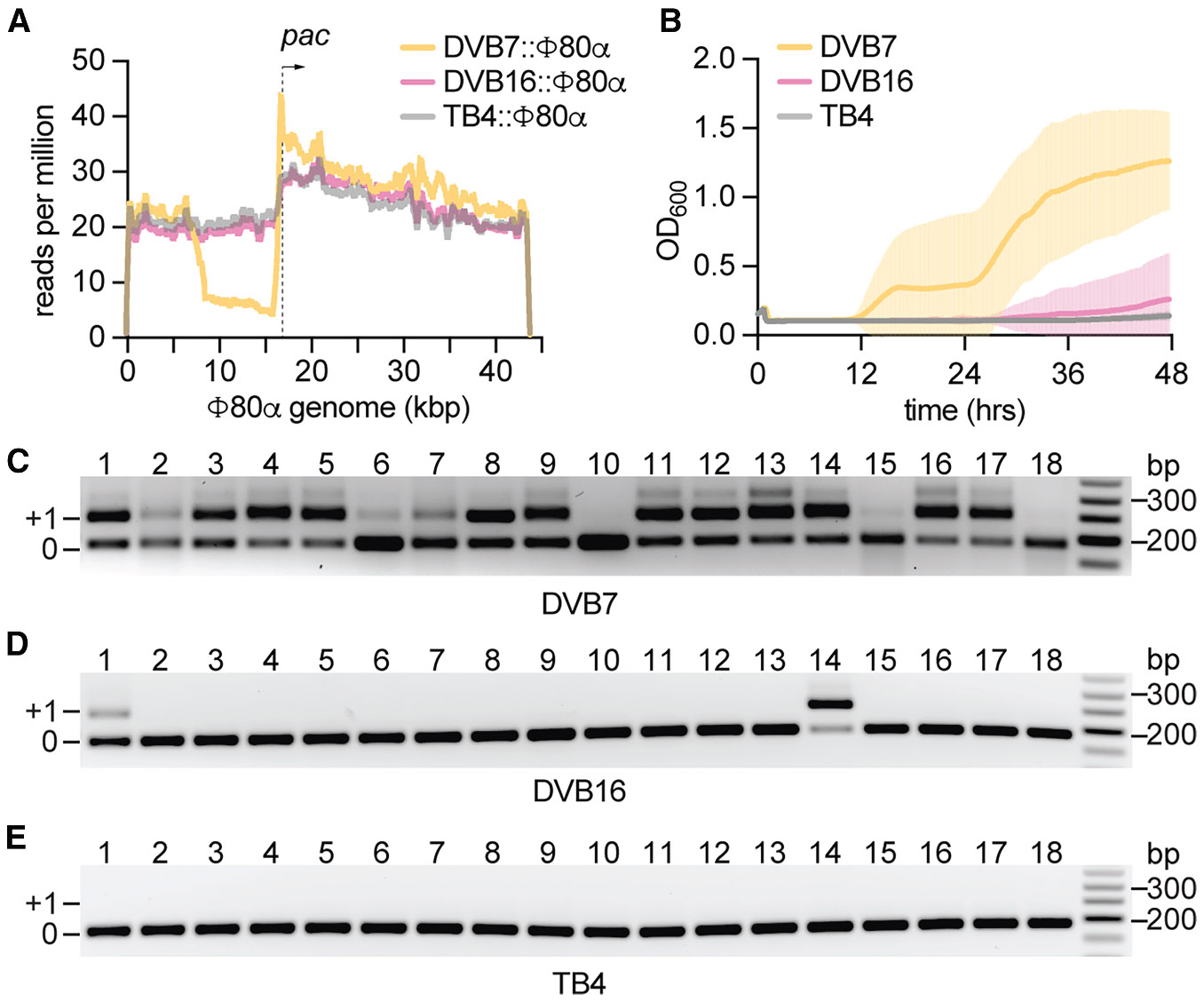
SaPI1-modified particles carrying partial helper phage genomes enhance spacer acquisition (A) NGS reads of DNA isolated from the supernatants of *S. aureus* DVB7:Φ80α, DVB16:Φ80α, or TB4:Φ80α lysogenic strains, obtained after mitomycin C induction, mapped along the Φ80α genome. The direction of Φ80α DNA packaging is denoted with an arrow initiating at a specific sequence motif known as the *pac* site. Data shown are the average of two biological replicates. (B) Growth of *S. aureus* TB4/pCRISPR-II measured at OD_600_ after infection with the lysates described in Figure S3A. The mean ± SD for 18 biological replicates is shown. (C) Detection of spacer acquisition within the type II-A CRISPR locus via PCR. Amplification products obtained using template DNA extracted from 18 *S. aureus* TB4/pCRISPR-II cultures treated with DVB7 (SaPI+) infection lysates were separated through agarose gel electrophoresis. Products indicated as “+1” show expansion of the CRISPR array with an additional repeat-spacer unit. “0” shows PCR products without such expansion. Molecular weight markers are shown on the right. (D) Same as (C) but using DNA extracted from 18 TB4/pCRISPR-II cultures treated with DVB16 infection lysates. (E) Same as (C) but using DNA extracted from 18 TB4/pCRISPR-II cultures treated with TB4 infection lysates. See also Figure S3

The results described above suggest that SaPI1 can enhance spacer acquisition in the bacterial population. Recently, it has been proposed that this adaptive process requires unproductive phage infections that lead to the injection of “defective” viral DNA, including phage DNA cleaved by restriction endonucleases^50,51^ or Cas9.^52^ It is believed that the cytosolic viral DNA is required as the substrate for the Cas1-Cas2 integrase complex, and the absence of a lytic cycle allows for both the acquisition process as well as the survival of the cell harboring the new spacers. Given that SaPI1 induction generates particles that contain Φ80α DNA,^33^ we hypothesized that SaPI1 particles harboring partial phage genomes are released upon host lysis and inject “defective” viral DNA into new host cells to enhance CRISPR spacer acquisition. To accurately determine the extent to which the Φ80α genome is packaged into SaPI1-modified capsids, we performed next-generation sequencing (NGS) of the DNA extracted from lysates obtained after mitomycin C induction of strains harboring the Φ80α helper prophage and SaPI1 (Figure 2A). We found that read coverage for ~35 kb of the Φ80α genome, with a sharp start at the *pac* site and a depletion of reads in a ~10 kb region immediately upstream of this site. This region is the last to be packaged into phage capsids^53^ and is therefore likely also last during packaging of Φ80α DNA into SaPI1-modified capsids; thus, this region is more often excluded due to the smaller size of these capsids. To confirm this, we abrogated the redirection of phage capsid assembly to form small capsids through deletion of the *cpmAB* operon^32^ (strain DVB16 [TB4:SaPI1 *tst::tetM* Δ*cpmAB*]). The NGS of the DNA present in the lysate of this culture resulted in reads covering the complete Φ80α genome, with a pattern nearly identical to that obtained after sequencing the DNA present in the lysate of Φ80α lysogens that lack SaPI1 (Figure 2A). These data corroborate that SaPI1-mediated capsid size redirection is responsible for the production of viral-like particles harboring partial phage genomes.

To directly test whether these defective particles enhance spacer acquisition, we obtained lysates after infection of TB4 (no SaPI1), DVB7 (carrying SaPI1), and DVB16 (harboring SaPI1 Δ*cpmAB*) with Φ80α-vir (Figure S3A). All of these lysates contain varying amounts of phage particles; therefore, we normalized their PFU titer before infecting 18 independent cultures of TB4/pCRISPR-II staphylococci to measure adaptive immunity in the absence of SaPI1 induction within the hosts. Only infection with the DVB7 lysate, which, according to our deep sequencing data shown in Figure 2A, contains defective particles with partial phage genomes, enabled a consistent re-growth of the cultures (Figures 2B and S3B), as well as spacer acquisition across independent replicates (Figure 2C). In contrast, the DVB16 and TB4 lysates lacking these defective Φ80α-vir particles did not promote the survival of most of the infected cultures (Figures 2B and S3C), which showed low rates of spacer acquisition (Figures 2D and 2E), and therefore failed to induce the type II-A CRISPR adaptive immunity. To further corroborate these results, we generated a lysate with SaPI1 particles that contain only this element’s DNA, not helper phage DNA. This lysate was obtained after mitomycin C induction of strain ST16, which harbors an Φ80α prophage lacking *terS*, the gene required for the packaging of the phage genome into the SaPI1-modified particle (RN4220:SaPI1 *tst::tetM*::Φ80α Δ*terS*).^33^ We then mixed the TB4 lysate (containing only Φ80α-vir) with pure SaPI1 particles at a concentration equal to that detected in DVB7 lysates. In this way, we generated a lysate comparable to that collected from DVB7 cells, but without the initial presence of “defective” phage particles for the first rounds of infection. After several lytic cycles, it is possible that some cells are co-infected first by SaPI1 and then by Φ80α-vir, and thereafter generate SaPI1-modified particles filled with viral DNA that immunize other surviving cells. Indeed, this lysate prevented the growth of TB4/pCRISPR-II staphylococci initially, but enabled some slow recovery 36 h post-infection (Figure S3D). Taken together, these results indicate that SaPI1-mediated injection of inactive partial phage genomes is fundamental for the enhancement of type II-A immunity.

### Type II-A CRISPR-Cas spacer diversity and SaPI1-mediated capsid size redirection prevent the propagation of both helper phages and SaPI1

**Figure 3.**
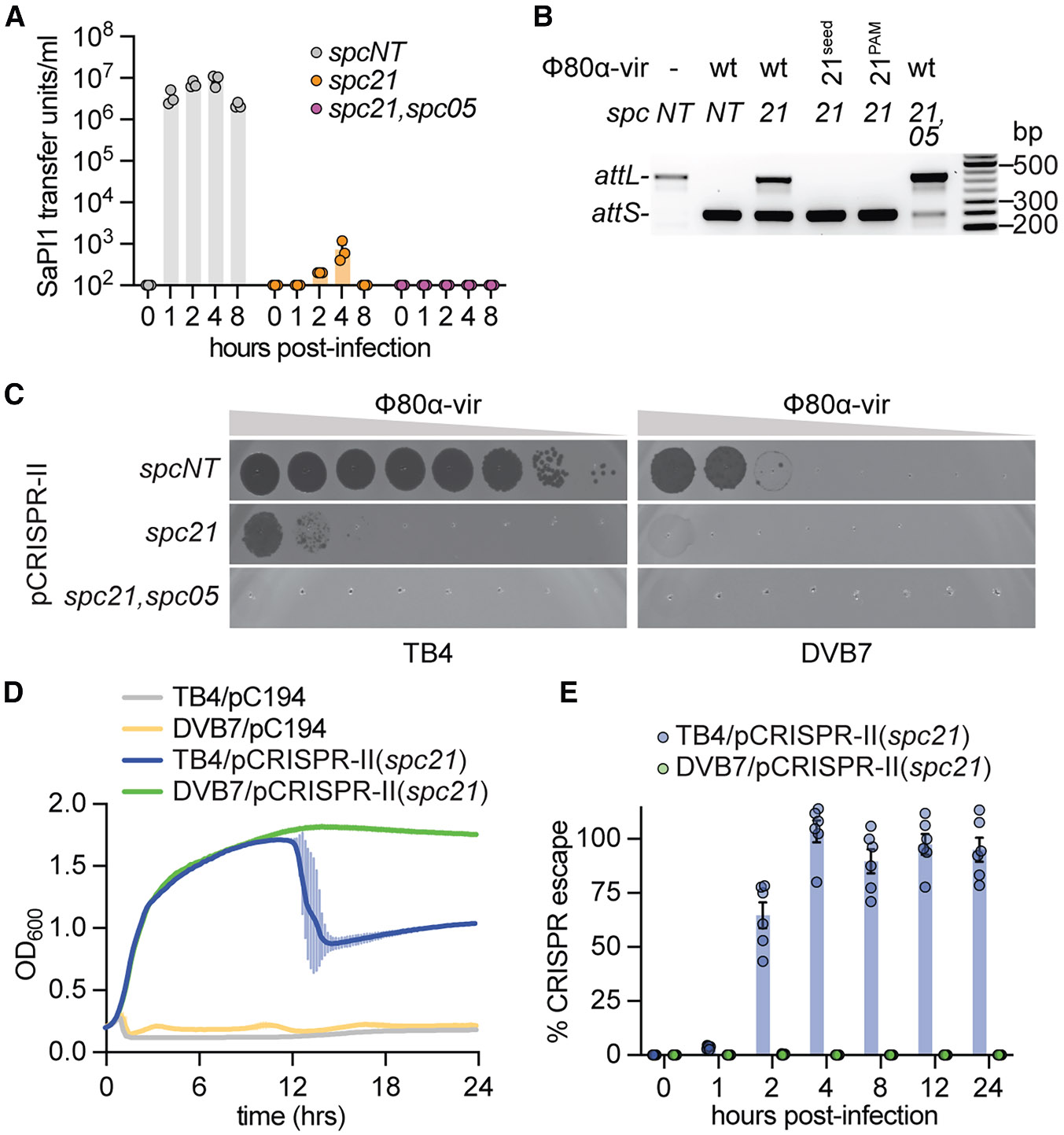
Type II-A CRISPR-Cas spacer diversity and SaPI1-mediated capsid size redirection prevent the propagation of both helper phages and SaPI1 (A) Enumeration of SaPI1 transfer units per milliliter of filtered supernatants taken at increasing times after Φ80α-vir infection of *S. aureus* DVB7 (SaPI+) cultures harboring pCRISPR-II plasmids programmed with different spacer sequences. The mean ± SEM of 3 biological replicates is shown. (B) Detection of the integrated (*attL*) or excised (*attS*) forms of SaPI1 via multiplex PCR 4 h after infection. Amplification products were obtained using template DNA extracted from *S. aureus* DVB7 (SaPI+) cultures harboring pCRISPR-II plasmids programmed with different spacer sequences and infected at MOI 10 with Φ80α-vir, either wild-type or harboring mutations that evade Cas9(*spc21*) targeting, and were separated through agarose gel electrophoresis. Molecular weight markers are shown on the right. (C) Detection of plaque formation after seeding 10-fold dilutions of Φ80α-vir on lawns of TB4 (SaPI−) or DVB7 (SaPI+) staphylococci harboring pCRISPR-II plasmids programmed with different spacer sequences. (D) Growth of TB4 (SaPI−) or DVB7 (SaPI+) *S. aureus* strains with or without pCRISPR-II(*spc21*), measured at OD_600_ after infection with Φ80α-vir at MOI 10. The mean ± SD for 3 biological replicates is shown. (E) Fraction (%) of phages that escape pCRISPR-II(*spc21*) targeting, obtained at different times after infection of *S. aureus* TB4/pCRISPR-II(*spc21*) or DVB7/pCRISPR-II(*spc21*) with Φ80α-vir at MOI 10. Mean ± SEM of 6 biological replicates is shown. See also Figure S4.

As demonstrated above, the infection of staphylococci harboring SaPI1 and a naive type II-A CRISPR-Cas locus leads to the generation of a phage-resistant bacterial population harboring both elements. Cells in this population contain a CRISPR locus that is programmed with a spacer that targets the helper phage, which should prevent SaPI1 induction if the culture is subsequently challenged with these viruses. We tested this prediction by infecting with Φ80α-vir DVB7/pCRISPR-II staphylococci carrying a plasmid targeting *gp21* (pCRISPR-II[*spc21*]) or a non-targeting spacer control (pCRISPR-II[*spcNT*)]) (Figures S4A and S4B). We then monitored SaPI1 transfer through a transduction assay at different times post-infection (Figure 3A). We observed that while many SaPI1 particles were released in the absence of CRISPR-Cas9 targeting, CRISPR immunity strongly reduced SaPI transfer with minimal, but still detectable transduction measured at the 2- and 4-h time points. This result suggests that SaPI1 is induced in a fraction of staphylococci harboring a type II-A CRISPR-Cas system targeting the helper phage. Therefore, we decided to directly examine SaPI induction via PCR, through the amplification of either the *attL* or *attS* sites, to detect integrated or episomal forms of SaPI1, respectively (Figure S4C). In the absence of helper phage infection, lack of induction resulted in the exclusive amplification of the *attL* integration site, only demonstrating stable integration of SaPI1 (Figure 3B). Conversely, after infection of staphylococci carrying pCRISPR-II(*spcNT*), high levels of SaPI1 induction led to the detection of the *attS* PCR product only. Consistent with the transduction results, we also observed some SaPI1 excision in the presence of pCRISPR-II(*spc21*). We reasoned that low levels of SaPI1 induction and transduction could be mediated by escaper phages containing target mutations that avoid type II-A targeting and express Sri. It has been shown that because a given phage is likely to evolve mutations in one, but not many, protospacers, spacer diversity leads to the neutralization of CRISPR escaper phages on the population level.^52,54^ Therefore, we measured SaPI1 induction in the presence of a CRISPR array harboring a second targeting spacer, *spc05* (Figure S4A). As opposed to staphylococci harboring only pCRISPR-II(*spc21*), infection of DVB7/pCRISPR-II(*spc21-spc05*) cells with Φ80α-vir substantially decreased SaPI1 induction (Figure 3B) and thus prevented SaPI1 transduction (Figure 3A). This result suggests that the low but detectable level of SaPI1 transduction and induction observed in the presence of pCRISPR-II(*spc21*) in Figures 3A and 3B, respectively, is mediated by helper phages that escape *spc21* targeting. Since these phages can be eliminated by the presence of additional spacers, the result also implies that the continued stimulation of the type II-A CRISPR-Cas response mediated by SaPI1 induction from infection by CRISPR escaper phages would further diversify the repertoire of spacers within the bacterial population^54^ and prevent the rise of escaper helper phages, thus restricting further SaPI1 induction.

Interestingly, although the above results suggest that escaper helper phages should be present in DVB7/pCRISPR-II(*spc21*) cultures, we found that Φ80α-vir could not form any plaques on the lawns of this strain (Figure 3C). However, the helper phage generated escaper plaques on lawns of TB4/pCRISPR-II(*spc21*) and TB4/pCRISPR-II(*spc05*) (Figure S4D), but as expected, not on staphylococci harboring pCRISPR-II(*spc21-spc05*) (Figure 3C). Isolation and sequencing of these *spc21*-escaper phages revealed the presence of mutations in either the seed sequence or PAM of the *spc21* target known to prevent type II-A CRISPR immunity^55^ (Figure S4E). We obtained similar results during the infection of liquid cultures. While TB4/pCRISPR-II(*spc21*) cultures treated with Φ80α-vir at MOI 10 succumb to infection after an initial period of growth, DVB7/pCRISPR-II(*spc21*) cultures are completely immune to infection (Figure 3D). We measured the fraction of escaper phages present in these cultures at different times during infection (Figures 3E and S4F) and found that mutant viruses rapidly propagated on TB4/pCRISPR-II(*spc21*) staphylococci, most likely causing the observed lysis of this culture, but were undetectable in DVB7/pCRISPR-II(*spc21*) cultures. Phage stocks of two of these escapers, Φ80α-vir(21^seed^) and Φ80α-vir(21^PAM^), can propagate on TB4/pCRISPR-II(*spc21*), but not DVB7/pCRISPR-II(*spc21*) staphylococci (Figure S4G). These CRISPR escapers also promote SaPI1 excision (Figure 3B) and transduction (Figure S4H) after infection of DVB7/pCRISPR-II(*spc21*) cultures. Therefore, we believe that these escaper helper phages are completely neutralized by capsid size redirection when they constitute a minimal fraction of the phage population. Altogether, these results demonstrate that after SaPI1 induction triggers the type II-A CRISPR-Cas adaptive immune response, both the spacer diversity of the population, as well as the continuing inhibition of viral propagation by SaPI1-mediated capsid size redirection, together contain the emergence and spread of escaper helper phages, preventing further SaPI1 mobilization.

### SaPI1 induction enhances spacer acquisition during the type III-A CRISPR-Cas response

**Figure 4.**
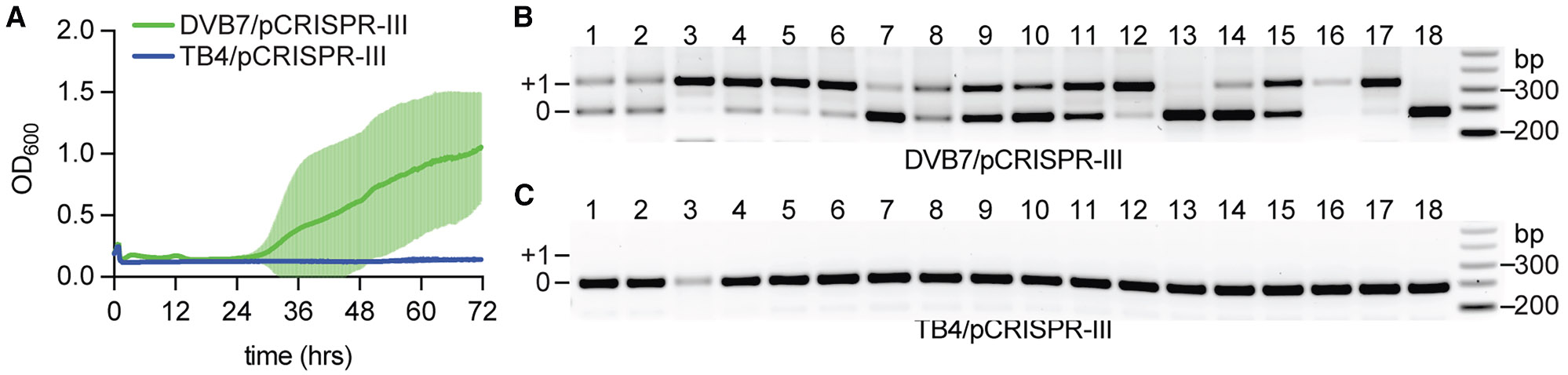
SaPI1 induction enhances spacer acquisition during the type III-A CRISPR-Cas response (A) Growth of *S. aureus* DVB7 (SaPI+) or TB4 (SaPI− ) strains harboring pCRISPR-III, measured at 600nm (OD_600_) after infection with Φ80α-vir at MOI 10. The mean ± SD for 18 biological replicates is shown. (B) Detection of spacer acquisition within the type III-A CRISPR locus via PCR. Amplification products obtained using template DNA extracted from 18 infected *S. aureus* DVB7/pCRISPR-III cultures, shown in (A), were separated through agarose gel electrophoresis. Products indicated as “+1” show expansion of the CRISPR array with an additional repeat-spacer unit. “0” shows PCR products without such expansion. Molecular weight markers are shown on the right. (C) Same as (B) but using DNA extracted from infected *S. aureus* TB4/pCRISPR-III cultures. See also Figure S5.

Besides type II-A CRISPR-Cas loci, staphylococci commonly carry type III-A systems that co-exist with PICIs (Table S1). To investigate the interplay between these two elements, we first determined the effect of SaPI1 on spacer acquisition into the *S. epidermidis* RP62A type III-A CRISPR locus (Figure S5A). To do this, we cloned this system harboring a single, non-targeting spacer into the pC194 vector to generate pCRISPR-III(*spcNT*)^28^ (Figure S5A), which we transformed into both TB4 and DVB7 strains. Similar to the type II-A system, only the SaPI1-containing cultures survived phage infection (Figure 4A), with robust regrowth in 15 out of the 18 independent experiments (Figure S5B). PCR analysis of the type III-A CRISPR locus (Figure S5A) in these 18 cultures showed spacer acquisition in 16 of them (Figure 4B), with the two that did not show expansion of the array (#13 and 18) being two of the three cultures that did not overcome phage infection (Figure S5B). Sequencing of the new spacers harbored by individual colonies obtained after plating these cultures corroborated spacer acquisition from the Φ80α-vir helper phage (Figure S5C). With one exception, all spacers sequenced mediated targeting of early-expressed transcripts, since these provide a positive selection for the cells that acquire them.^56^ In contrast, TB4/pCRISPR-III(*spcNT*) cultures succumbed to Φ80α-vir infection (Figure 4A) and did not acquire spacers (Figure 4C), a result that is in agreement with the low frequency of spacer acquisition of type III systems, a much rarer event than for type II CRISPR loci.^56-58^ The detection of new phage-derived spacers despite the inefficiency of type III CRISPR loci to incorporate new spacers underscores injection of partial phage genomes mediated by SaPI1 remodeling of viral capsids as a general and highly efficient mechanism to enhance CRISPR immunization.

### Activation of the Csm6 RNase during the type III-A CRISPR-Cas response prevents SaPI1 mobilization

**Figure 5.**
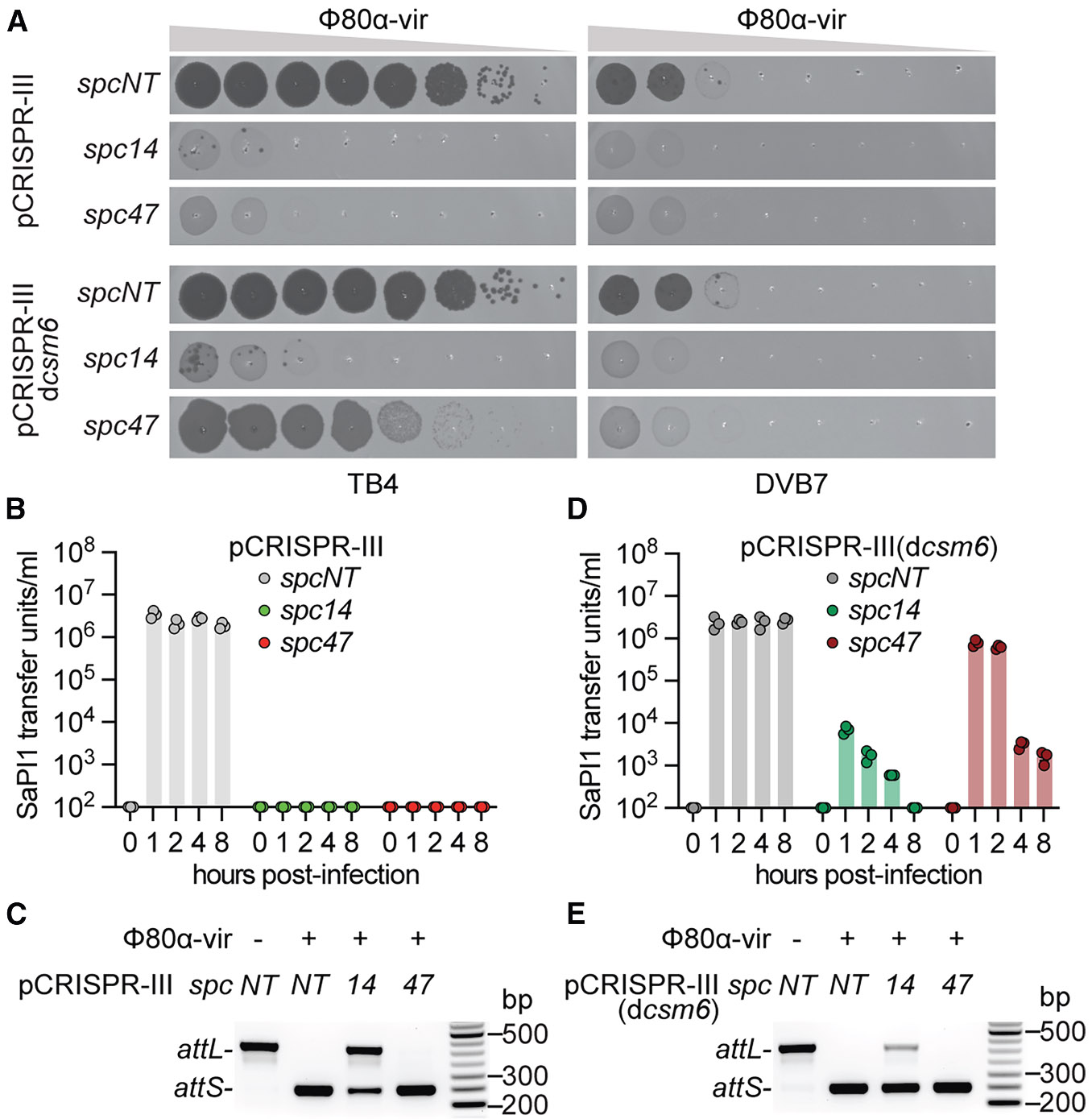
Activation of the Csm6 RNase during the type III-A CRISPR-Cas response prevents SaPI1 mobilization (A) Detection of plaque formation after seeding 10-fold dilutions of Φ80α-vir on lawns of TB4 (SaPI−) or DVB7 (SaPI+) staphylococci harboring pCRISPR-III or pCRISPR-III(d*csm6*) plasmids programmed with different spacer sequences. (B) Enumeration of SaPI1 transfer units per milliliter of filtered supernatants taken at increasing times after infection of *S. aureus* DVB7 (SaPI+) cultures harboring pCRISPR-III plasmids programmed with different spacer sequences. The mean ± SEM of 3 biological replicates is shown. (C) Detection of the integrated (*attL*) or excised (*attS*) forms of SaPI1 via multiplex PCR 4 h after infection. Amplification products were obtained using template DNA extracted from *S. aureus* DVB7 (SaPI+) cultures harboring pCRISPR-III plasmids programmed with different spacer sequences and infected with Φ80α-vir at MOI 10, and were separated through agarose gel electrophoresis. Molecular weight markers are shown on the right. (D) Same as (B) but after infection of *S. aureus* DVB7 (SaPI+) cultures harboring pCRISPR-III(d*csm6*) plasmids programmed with different spacer sequences. The mean ± SEM of 3 biological replicates is shown. (E) Same as (C) but using DNA extracted from infected *S. aureus* DVB7 (SaPI+) cultures harboring pCRISPR-III(d*csm6*) plasmids programmed with different spacer sequences. See also Figure S6.

Given that the initial round of SaPI1 mobilization results in the reprogramming of the type III-A CRISPR-Cas defense against the helper phage, we decided to study how this immunity impacts further SaPI1 spread. To do this, we used two spacers, *spc14* and *spc47*, which target the early- and late-expressed genes *gp14* and *gp47*, respectively. The immunity provided by these spacers against the related phage ΦNM1g6^59^ was previously characterized.^28,60^ It was found that the defense mediated by crRNAs complementary to an early-expressed transcript, such as *gp14*, relies on the DNase activity of Cas10 and results in the destruction of the viral genome and the survival of the infected cell. In contrast, targeting of a late-transcribed phage mRNA, such as *gp47*, depends on Csm6, a non-specific RNase that degrades cellular transcripts indiscriminately to induce the dormancy of the infected host and prevent viral propagation.^29^ First, we studied the effect of pCRISPR-III(*spc14*) targeting Φ80α-vir on SaPI1 induction, which mediates a mechanism of immunity similar to pCRISPR-II(*spc21*); i.e., a nucleolytic attack on the viral DNA early during the lytic cycle. Plaque assays showed that while rare phage escapers of *spc14* targeting were present in TB4 lawns, they were not detected when we infected DVB7 cells (Figure 5A). These data suggest that, similar to the results for type II-A immunity, SaPI1 induction results in the neutralization of helper viruses that escape Cas10-mediated DNA degradation via capsid size redirection. In contrast, while SaPI1 induction by Φ80α-vir phages that evade Cas9 targeting leads to low levels of island transduction (Figure 3A), we were unable to detect transducing particles in the supernatants of infected DVB7/pCRISPR-III(*spc14*) cultures (Figure 5B). Surprisingly, PCR analysis of the infected cultures showed considerable SaPI1 excision, presumably triggered by escaper helper phages (Figure 5C). We hypothesized that Csm6 RNase activity could affect the completion of the SaPI1 life cycle after it is induced by these escapers. Although not required for immunity (Figure 5A), this non-specific RNase activity is triggered during the type III-A response mediated by *spc14* and could degrade SaPI1 transcripts. To test this, we measured SaPI1 excision and transduction in DVB7/pCRISPR-III(*spc14*, d*csm6*) cultures, which carry a mutation in the Csm6 active site.^28^ These results are equivalent to those obtained after infection of DVB7/pCRISPR-II(*spc21*) cultures; i.e., intermediate levels of SaPI1 transduction (Figure 5D) and excision (Figure 5E). Therefore, we conclude that the activation of Csm6 during *spc14* targeting of Φ80α-vir prevents the production and subsequent dissemination of SaPI1 particles.

We wondered how targeting of the helper phage through *spc47*, which mediates a type III-A response triggered late in the lytic cycle that is entirely dependent on Csm6 activity, would affect SaPI1 transduction. We first performed plaque assays on lawns of TB4 or DVB7 staphylococci carrying pCRISPR-III(*spc47*) (Figure 5A), which showed complete inhibition of phage propagation. Therefore, as opposed to previous experiments with type II-A and *spc14*-mediated type III-A targeting, SaPI1 is not required to prevent the rise of helper phages that escape *spc47*-mediated type III-A targeting. This observation aligns with previous reports demonstrating the ability of Csm6 to neutralize phages with target mutations^28,61^ and correlated with the lack of detection of SaPI1 transduction (Figure 5B). However, despite the absence of escaper helper phages, we unexpectedly observed complete induction of SaPI1 via PCR (Figure 5C). These high levels of SaPI1 excision are likely due to the transcription-dependent activation of the type III-A CRISPR-Cas response,^59^ which, when mediated by *spc47*, occurs after the transcription of the SaPI1 inducer *sri* (*gp22*, Figure S4A). Later in the Φ80α-vir lytic cycle, activation of the Csm6 RNase leads to the generation of an inhospitable cell for the propagation of not only the helper phage, but also SaPI1. This was corroborated by experiments performed in staphylococci lacking Csm6 RNase activity, carrying pCRISPR-III(*spc47* and d*csm6*). Consistent with previous results,^28^ type III-A immunity failed to restrict Φ80α-vir plaque formation in the absence of this RNase; however, the presence of SaPI1 restored defense (Figure 5A), presumably through interference of the lytic cycle caused by capsid redirection. The lack of immunity against the helper phage in this mutant correlated with substantial SaPI1 transduction (Figure 5D), as well as with complete excision of this element (Figure 5E) after infection. Interestingly, the increase in SaPI transduction observed in staphylococci expressing Csm6 during *spc47* targeting decreased with time, as opposed to the results obtained after infection of non-targeting cells (Figure 5D). This suggests that the non-specific single-strand DNAse activity of Cas10, although not sufficient to provide detectable immunity against Φ80α-vir (Figure 5A), can interfere with SaPI1 function.

RNA targeting by the Cas10-Csm complex tolerates the establishment of lysogeny when the target transcript is silenced in the integrated prophage.^59^ Many staphylococcal clinical isolates contain multiple prophages in addition to SaPIs,^62^ which in many cases can act as helper phages when induced.^15^ Therefore, we decided to investigate the effects of helper prophage induction on SaPI1 excision and transduction in the presence of type III-A CRISPR-Cas immunity. To do this, we generated a derivative of strain TB4 harboring both the ΦNM1 prophage^41^ (Figure S6A) and SaPI1, named DVB3, which was then transformed with different pCRISPR-III plasmids programmed with spacers that target early- and late-expressed transcripts from this helper phage (*spc19* and *spc43*, respectively, Figure S6B), as well as a non-targeting control. Using these strains, we measured SaPI1 excision and transduction upon treatment of bacterial cultures with mitomycin C to induce ΦNM1. Similar to the results obtained after infection with the lytic helper phage Φ80α-vir, type III-A targeting of the early-expressed viral transcripts during induction of the helper prophage ΦNM1 prevented both SaPI1 excision (Figure S6C, *spc19*) and transduction (Figure S6D, *spc19*). Targeting of late-expressed phage transcripts still prevented SaPI1 transfer (Figure S6D, *spc43*) despite full excision of the island (Figure S6C, *spc43*). This is likely mediated by indiscriminate RNA cleavage by Csm6 during type III-A immunity, as was the case during Φ80α-vir infection. Indeed, when the RNase activity of Csm6 was absent, restriction on SaPI1 transfer is decreased (Figure S6D, *dcsm6*), to a greater extent during targeting of the *gp43* transcript than of the *gp19* RNA.

Altogether, these results demonstrate that, similar to type II-A CRISPR-Cas systems, the type III-A adaptive immune response against helper phages, once stimulated by SaPI1 induction, prevents further spread of this element. However, in contrast to type II-A immunity, which requires spacer diversity to prevent further SaPI1 induction by Φ80α-vir escapers, the Csm6 RNase activity of type III-A systems is sufficient to counteract the rise of escapers and limit additional SaPI1 transduction.

## DISCUSSION

Here, we investigated the complex interplay between CRISPR-Cas systems, SaPIs, and their helper phages in staphylococci. We discovered an unexpected synergy between CRISPR and SaPIs: the enhancement of acquisition of spacer sequences from the helper phage genome following SaPI1 induction. We propose that this phenomenon is mediated by the generation of SaPI1 particles containing partial Φ80α-vir genomes.^33^ In a process akin to vaccination with attenuated pathogens that provide antigens for the generation of memory antibodies during the mammalian adaptive immune response,^63^ these particles inject non-infective phage DNA that can be used as a source of spacers for the CRISPR acquisition machinery^64,65^ in order to generate a memory of infection. This is a new mechanism that generates non-functional viral DNA within the host for the acquisition of new spacers, different from the cleavage of the injected phage genome by restriction enzymes^50^ or Cas9,^52^ or bacteriostatic antibiotics that generate dormant hosts and thus disrupt the viral lytic cycle.^66^ Furthermore, capsid size redirection is a conserved, widespread strategy among different viral satellites,^21,67^ suggesting that our findings may extend to other PICIs and PLEs in a diverse range of bacteria harboring CRISPR-Cas systems.

After promoting spacer acquisition throughout the population, SaPI1 loses its ability to further spread to new hosts, as the CRISPR-Cas systems are now programmed to target and destroy the helper phage. For type II-A CRISPR immunity, in principle, escaper phages with target mutations that bypass Cas9 targeting^55,68^ could theoretically spread and restart another cycle of SaPI1 induction and spread. However, we found two mechanisms that prevent this. First, we observed another synergy between type II-A CRISPR and SaPI1: mutant phages that are not cleaved by Cas9 can induce SaPI1, which prevents their propagation through capsid size redirection.^20^ Second, the high diversity of spacers within the CRISPR-adapted bacterial population also enables the neutralization of escaper phages.^52,54^ In contrast to these mechanisms, during the type III-A CRISPR-Cas response, Csm6 RNase activity^29^ prevents the propagation of escapers. It is believed that, similar to the RNase activity of Cas13a during type VI-A CRISPR immunity,^69^ the minority of mutant phages that can escape targeting and lyse the host eventually, end up infecting a dormant cell in which the Csm6 RNase was activated by a wild-type phage; i.e., enter a compromised host that cannot support their propagation. In addition to hindering the proliferation of escaper helper phages, it is also possible for Csm6 to limit SaPI1 spread through the degradation of transcripts essential for the generation of transducing particles.

### Limitations of the study

Our data showed that through distinct mechanisms, both type II-A and III-A CRISPR systems of staphylococci limit SaPI1 transduction. We reached this conclusion working with a simplified experimental system, using a laboratory staphylococcal strain that lacks additional defense systems that target the helper phage, prophages, conjugative plasmids, and other mobile genetic elements. The absence of these multiple variables facilitated the interpretation of the results. Staphylococci in nature, however, are known to harbor simultaneously multiple mobile genetic elements, many of which encode immune systems that could potentially influence the interactions between CRISPR immunity and SaPI induction and transduction. Examples of this are the discovery of phage defense systems carried by SaPIs,^22^ as well as the incorporation of CRISPR systems into *Vibrio cholerae* helper phages to target PLEs.^70^ Even with these limitations, it is interesting to speculate about how our findings could influence the evolution of SaPIs and staphylococci. First, for the individual host, CRISPR targeting of the helper phage prevents the lethality of viral infection and, at the same time, ensures the retention of the SaPI and the benefits it confers through vertical dissemination of the element among staphylococci. Second, at the population level, the restriction imposed by CRISPR-Cas immunity on the horizontal spread of SaPIs may carry evolutionary costs given the aforementioned benefits of the pathogenicity islands. Such costs have been observed during type III-A CRISPR-Cas against antibiotic-resistant, conjugative staphylococcal plasmids,^71^ and have fueled the hypothesis that the targeting of beneficial MGEs contributes to the sparse distribution of CRISPR-Cas in bacterial pathogens.^72^ This is indeed the case in *S. aureus* where, in contrast to SaPIs, which are ubiquitous,^73^ CRISPR-Cas systems are relatively rare, present in only 0.5% of the sequenced isolates of this species.^74^ However, given the multiple elements involved in horizontal gene transfer in staphylococci, although it is tempting to speculate that the spread of advantageous MGEs like SaPIs and plasmids is favored over CRISPR systems in staphylococci, the actual evolutionary drivers in native environments remain unclear due to the presence of intricate and often counteracting forces that are still uncharacterized or even unknown and will constitute the premise of future studies using clinical isolates.

## STAR★METHODS

### EXPERIMENTAL MODEL AND STUDY PARTICIPANT DETAILS

#### Bacterial strains

The bacterial strains used in this study are listed in Table S2 with details of their construction. *Staphylococcus aureus* RN4220,^75^ TB4,^41^ and their derivative strains were grown at 37° C in brain heart infusion (BHI) broth or agar (BD Difco), with shaking at 220 RPM for liquid cultures. Wherever applicable, growth media were supplemented with chloramphenicol (10 μg mL^−1^) to maintain pC194-based plasmids,^46^ tetracycline (2.5 μg mL^−1^) to select for SaPI1 *tst::tetM*-harboring strains, or erythromycin (10 μg mL^−1^) to select for OS2, a variant strain of RN4220 with a chromosomal insertion of *ermC* into *spa*.^76^

#### Bacteriophages

The bacteriophages used in this study are listed in Table S3. To generate a high-titer phage stock, an overnight culture of *S. aureus* RN4220 was diluted 1:100 and grown to mid-log phase (~90 min) in BHI broth supplemented with 5 mM CaCl_2_. The culture was diluted to an optical density measurement at 600 nm (OD_600_) of 0.5 (1 × 10^8^ CFU mL^−1^). The culture was infected by adding phage at a multiplicity of infection (MOI) of 0.1 (1 × 10^7^ PFU mL^−1^), or by inoculating with either a single picked plaque or scrape of a frozen stock. The infected culture was grown at 37° C with shaking and monitored for lysis (full loss of turbidity was typically observed ~3–4 h). Culture lysates were centrifugated (10,000 x g for 10 min) to pellet cellular debris. The supernatant was collected, passed through a sterile membrane filter (0.45 μm), and stored at 4° C for short-term use. Phage titers were determined by serially diluting the obtained stock in 10-fold increments and spotting 5 μL of each dilution on BHI soft agar mixed with RN4220 supplemented with 5 mM CaCl_2_. After incubation overnight at 37° C, individual plaques (i.e., zones of no bacterial growth) were counted, and the viral titer represented by plaque-forming units (PFU) per milliliter was calculated.

### METHOD DETAILS

#### Molecular cloning

The plasmids (including details of their construction) and the oligonucleotide primers used in this study are listed in Tables S4 and S5, respectively. For insertion of a desired spacer sequence into the pCRISPR array, 1 μg of plasmid DNA was digested with 1 unit of BsaI-HF restriction enzyme (NEB) at 37° C for 2 h. Primers corresponding to the top and bottom strand of the spacer sequence were annealed by incubating the reaction mix (3 mM of each primer in nuclease-free water and 50 mM NaCl) at 95° C for 5 min on a heat block, which was then removed from heat to gradually cool to room temperature. Annealed oligonucleotides were ligated to BsaI-digested plasmids by incubating the 15 μL reaction mix (10 μL digested plasmid, 2 μL of 1:10 dilution of annealed oligos, 1.5 μL T4 DNA Ligase 10x Buffer, 0.5 μL T4 DNA Ligase) at room temperature for 2 h. For Gibson assembly,^78^ double-stranded DNA fragments in nuclease-free water were combined in equimolar ratios to a total volume of 5 μL. Reaction mixtures were combined with 15 μL Gibson assembly master mix (composed of T5 exonuclease, Phusion High-Fidelity DNA Polymerase, and Taq ligase in ISO Buffer) and incubated at 50° C for 1 h. Electrocompetent *S. aureus* RN4220 or TB4 cells (prepared through several rounds of washing with 0.5 M sucrose, as previously described^79^) were transformed by mixing 5 μL of the drop-dialyzed ligation or Gibson assembly products into 50 μL of cells in a 0.2 cm gap cuvette and pulsed once using a Bio-Rad MicroPulser (settings: 2900 V, 25 μF, 100 Ω, with typical time constant of 2.5–2.7 ms). Cells were resuspended in 950 μL of BHI without selective antibiotics, recovered for 1 h shaking at 37° C, and plated onto BHI agar containing the appropriate antibiotic(s) for selection.

#### Isolation of escaper bacteriophages

High-titer phage lysates were spotted onto soft agar lawns of *S. aureus* TB4 harboring SaPI1 (DVB7) or pCRISPR-II(*spc21*). Individual plaques were picked and resuspended in 30 μL of BHI broth. Sanger sequencing of PCR-amplified target regions of Φ80α-vir was performed to identify the presence of any mutations. The escaper phages were then further purified by isolating single plaques over two rounds of passaging on the appropriate selective strain.

#### Soft agar phage infection

100 μL of an overnight bacterial culture supplemented with 5 mM CaCl_2_ was mixed into 5 mL BHI soft agar and poured on top of BHI agar plates to solidify at room temperature (~15 min). Phage lysates were serially diluted 10-fold and 2.5–5 μL was spotted onto the soft agar surface. Once dry, plates were incubated at 37° C overnight and visualized the next day. Individual plaques were counted manually.

#### Liquid culture phage infection or prophage induction

Overnight cultures were diluted 1:100 in BHI supplemented with 5 mM CaCl_2_ and the appropriate selective antibiotic, outgrown at 37° C with shaking to mid-log phase (~90 min), and normalized to OD_600_ 0.5 (1 × 10^8^ CFU mL^−1^). With infection, for the desired MOI, a calculated volume of phage stock or infection lysate was added to each culture. With prophage induction, cultures were supplemented with mitomycin C (AG Scientific) to a final concentration of 1 μg mL^−1^. 150 μL of this phage-infected or mitomycin C-induced culture was seeded into each well of a 96-well plate. OD_600_ was measured every 10 min in a microplate reader (TECAN Infinite 200 PRO) at 37° C with shaking. Bacterial titers were determined by serially diluting the culture in 10-fold increments and spotting 5 μL of each dilution on BHI agar. After incubation overnight at 37° C, individual colony-forming units (CFU) were counted, and bacterial titer represented by CFU per milliliter was calculated.

#### Time-shift assay

Overnight cultures of DVB7 were diluted 1:100 in BHI supplemented with 5 mM CaCl_2_ and outgrown at 37° C with shaking to mid-log phase (~90 min), and normalized to OD_600_ 0.5 (1 × 10^8^ CFU mL^−1^). Cultures were infected with Φ80α-vir at MOI 10 and samples were taken at the following timepoints: 30 min, 1 h, 90 min, 2 h, 3 h, and 4 h. Culture lysates were centrifugated (10,000 x g for 10 min) to pellet cellular debris. Supernatants were collected and the resulting phage lysates were serially diluted and spotted onto TB4 and DVB7 to measure phage titers. For each replicate sample, the fraction of SaPI1-escaper phages was calculated by dividing the phage titer obtained on DVB7 with the phage titer obtained on TB4.

#### SaPI titer measurement by transduction assay

Culture supernatants from phage infection experiments of strains harboring SaPI1 *tst::tetM* were collected and passed through a sterile membrane filter (0.45 μm). 10 μL of filtered lysate was mixed with 90 μL of an overnight culture of the marker strain OS2 supplemented with 5 mM CaCl_2_ and normalized to OD_600_ 5.0 (1 × 10^9^ CFU mL^−1^). The mixture was incubated at room temperature for 30 min, and then 2 μL of 2 M sodium citrate was added (to a final concentration of 40 mM) to stop phage adsorption. Cultures were recovered for 2 h at 37° C with shaking and then plated on BHI agar supplemented with tetracycline (2.5 μg mL^−1^), erythromycin (10 μg mL^−1^), and sodium citrate (20 mM). After incubation overnight at 37° C, individual colonies were counted, and the SaPI titer represented by SaPI transfer units (STU) per milliliter was calculated.

#### PCR assays for CRISPR spacer acquisition and SaPI1 excision-integration

1-5 μL of liquid cultures obtained after phage infection was mixed into colony lysis buffer (250 mM KCl, 50 mM Tris-HCl pH 9.0, 5 mM Cl_2_Mg ⋅ 6H_2_O, 0.5% Triton X-100) supplemented with lysostaphin (final concentration of 100 μg mL^−1^) to a total volume of 20 μL. This mixture was incubated at 37° C for 20 min and then 98° C for 10 min to disrupt bacterial membranes and release DNA. PCR amplification was then performed using Phusion High-Fidelity DNA Polymerase (Thermo Fisher Scientific). For CRISPR spacer acquisition experiments, the primer pairs oDVB225/oDVB420 for pCRISPR-II and oDVB658/oDVB659 for pCRISPR-III were used (to a final concentration of 0.5 μM for each primer). To differentially amplify the SaPI1 junctions *attL* and *attS* uniquely present in either the integrated or excised form, respectively, a primer cocktail consisting of oGG338 (0.375 μM), oGG340 (0.75 μM), and oGG341 (0.375 μM) was used.

#### Next-generation sequencing of Φ80α DNA in infection lysates

Overnight cultures of *S. aureus* TB4 lysogen strains harboring the helper prophage Φ80α (DVB6), Φ80α and SaPI1 *tst::tetM* (DVB8), or Φ80α and SaPI1 *tst::tetM* Δ*cpmAB* (DVB21) were diluted 1:100 in BHI and outgrown at 37° C with shaking for 60 min (to early-log phase). Cultures were then supplemented with mitomycin C (AG Scientific) to a final concentration of 1 μg mL^−1^ and allowed to grow until complete lysis (~3–4 h). Lysates were centrifuged (10,000 x g for 10 min) to remove cellular debris and the clarified supernatant was then passed through a sterile membrane filter (0.45 μm) and stored at 4° C. Genomic DNA was extracted from phage lysates using a previously described method.^80^ DNA was sheared to 300-bp fragments using an S220 Covaris Focused-Ultrasonicator (peak incident power: 140 W, duty factor: 10%, cycles per burst: 200, treatment time: 80 s, temperature 4° C) in an S-Series Holder microTUBE (PN 500114). Library preparation was performed using an Illumina TruSeq LT DNA Library Preparation Kit following the manufacturer’s protocol. 12 pM of the library was loaded on an Illumina MiSeq instrument for paired-end sequencing (2 × 150 cycles). Bowtie2 via the Galaxy open-source interface^81^ was used to align sequencing reads to the Φ80α genome. A custom Python script was used to convert the output SAM alignments into CSV files.

#### Genomic analyses of staphylococcal PICIs and CRISPR-Cas systems

*Staphylococcus* genomes carrying complete CRISPR-Cas systems were identified using CRISPRCasdb^37^ with the strictest inclusion criteria (CAS and CRISPR evidence 4). From this list of 111 candidate genomes, putative PICIs were then identified using a combination of manual curation and a recently developed bioinformatic tool for predicting PICIs and PICI-like elements called Satellite-Finder.^38^ A list of the analyzed staphylococcal strains, the type of CRISPR-Cas system(s) that each strain harbors, and whether there exist any predicted PICI elements, are documented in Table S1.

### QUANTIFICATION AND STATISTICAL ANALYSIS

GraphPad Prism 10.6.1 was used to perform all statistical tests and generate graphs from experimental data. Adobe Illustrator 29.7 was used to perform any additional graphical formatting during figure preparation. For each individual experiment, the figure legends provide a description of any statistical test used, dispersion and precision measures, the exact value of n, and what n represents.

## Supplementary Material

1

Supplemental information can be found online at https://doi.org/10.1016/j.celrep.2025.116776.

## Figures and Tables

**Table:** KEY RESOURCES TABLE

REAGENT or RESOURCE	SOURCE	IDENTIFIER
Bacterial and virus strains		
*S. aureus* RN4220	Kreiswirth et al.^75^	N/A
*S. aureus* RN10616	Ubeda et al.^33^	N/A
*S. aureus* RN10628	Ubeda et al.^33^	N/A
*S. aureus* OS2	Schneewind et al.^76^	N/A
*S. aureus* ST16	Ubeda et al.^33^	N/A
*S. aureus* ST24	Ubeda et al.^33^	N/A
*S. aureus* ST126	Damle et al.^77^	N/A
*S. aureus* TB4	Bae et al.^41^	N/A
*S. aureus* DVB3	This study	N/A
*S. aureus* DVB6	This study	N/A
*S. aureus* DVB7	This study	N/A
*S. aureus* DVB8	This study	N/A
*S. aureus* DVB11	This study	N/A
*S. aureus* DVB16	This study	N/A
*S. aureus* DVB21	This study	N/A
Φ80α-vir	Banh et al.^42^	N/A
Φ80α-vir SaPI1-escaper #1	This study	N/A
Φ80α-vir(21^seed^)	This study	N/A
Φ80α-vir(21^PAM^)	This study	N/A
Chemicals, peptides, and recombinant proteins		
Brain-Heart Infusion (BHI) Agar	Becton Dickson	Cat#241810
Brain-Heart Infusion (BHI) Broth	Becton Dickson	Cat#238400
Chloramphenicol	GoldBio	Cat#C-105-100
Erythromycin	GoldBio	Cat#E–350-100
Tetracycline Hydrochloride	GoldBio	Cat#T-101-100
Calcium Chloride Dihydrate (White Crystals to Powder), Fisher BioReagents	Fisher Scientific	Cat#BP510-500
Glycerol (Certified ACS), Fisher Chemical	Fisher Scientific	Cat#G33-500
T4 Polynucleotide Kinase	NEB	Cat#M0201S
T4 DNA Ligase	NEB	Cat#M0202M
T4 DNA Ligase Reaction Buffer	NEB	Cat#B0202S
BsaI-HF	NEB	Cat#R3535L
Mitomycin C	AG Scientific	Cat#M-1108
Phusion High-Fidelity DNA Polymerase	Thermo Fisher Scientific	Cat#F530L
Applied Biosystems Fast SYBR Green Master Mix	Thermo Fisher Scientific	Cat#4385612
NEBuilder HiFi DNA Assembly Master Mix	NEB	Cat#2621L
Lysostaphin	AMBI Products	Cat#LSPN-50
Critical commercial assays		
MinElute PCR Purification Kit	QIAGEN	Cat#28004
QIAprep Spin Miniprep Kit	QIAGEN	Cat#27104
QIAquick Gel Extraction Kit	QIAGEN	Cat#28704
DNeasy Blood & Tissue Kit	QIAGEN	Cat#69504
Wizard Genomic DNA Purification Kit	Promega	Cat#A1125
Qubit 1X dsDNA HS Assay Kit (Invitrogen)	Thermo Fisher Scientific	Cat#Q33231
High Sensitivity D1000 ScreenTape	Agilent	Cat#5067-5584
High Sensitivity D1000 Reagents	Agilent	Cat#5067-5585
TruSeq Nano DNA LT Library Prep Kit	Illumina	Cat#20015964
MiSeq Reagent Kit v2 (300-cycles)	Illumina	Cat#MS-102-2002
Deposited data		
Next-generation sequencing files (Illumina NGS)	Sequence Read Archives (SRA)	BioProject accession: PRJNA1357993
Oligonucleotides		
See “Oligonucleotide primers used in this study” in Table S5 for nucleotide sequences		N/A
Recombinant DNA		
See “Plasmids used in this study” in Table S4 for construction details		N/A
pC194	Horinouchi et al.^46^	N/A
pDVB08	Banh et al.^42^	N/A
pDVB47, designated pCRISPR-II(*spcNT*)	This study	N/A
pDVB52, designated pCRISPR-II(*spc21*)	This study	N/A
pDVB55, designated pCRISPR-II(*spc14*)	This study	N/A
pDVB59, designated pCRISPR-III(*spcNT, dcsm6*)	This study	N/A
pDVB61, designated pCRISPR-III(*spc14, dcsm6*)	This study	N/A
pDVB96, designated pCRISPR-II(*spc21,spc05*)	This study	N/A
pDVB99, designated pCRISPR-II	This study	N/A
pDVB119, designated pCRISPR-II(Δ*cas1*)	This study	N/A
pGG-BsaI-R, designated pCRISPR-III(*spcNT*)	Jiang et al.^28^	N/A
pWJ191, designated pCRISPR-III(*spc47*)	Jiang et al.^28^	N/A
pWJ241, designated pCRISPR-III(*spc47,dcsm6*)	Jiang et al.^28^	N/A
Software and algorithms		
Photoshop CC	Adobe	https://www.adobe.com/products/photoshop.html
Illustrator CC	Adobe	https://www.adobe.com/products/illustrator.html
SnapGene	Insightful Science	https://www.snapgene.com
Python v3.8	Python Software Foundation	https://www.python.org/downloads/release/python-380/
PyCharm CE	JetBrains	https://www.jetbrains.com/pycharm/download/#section=mac
GraphPad Prism 10	Insightful Science	https://www.graphpad.com/scientific-software/prism/
Custom code used to analyze NGS data by converting.sam files into.wig files	This study	https://github.com/Marraffini-Lab/Banh_etal_2025
Other		
Infinite 200 PRO Plate Reader	TECAN	N/A
MiSeq System	Illumina	N/A
S220 Focused-Ultrasonicator	Covaris	N/A
S-Series Holder microTUBE	Covaris	Cat#500114

## Highlights

- SaPIs and Type II-A and III-A CRISPR-Cas systems co-occur in staphylococci
- SaPIs package partial genomes of helper phages that induce their replication
- Injection of incomplete phage genomes by SaPIs enhances the rate of CRISPR immunization
- Immunized hosts are protected from further infection by helper phages, favoring SaPI retention

## References

[1] Makarova KS, Wolf YI, Iranzo J, Shmakov SA, Alkhnbashi OS, Brouns SJJ, Charpentier E, Cheng D, Haft DH, Horvath P, (2020). Evolutionary classification of CRISPR-Cas systems: a burst of class 2 and derived variants. Nat. Rev. Microbiol 18, 67–83. 10.1038/s41579-019-0299-x. 31857715 PMC8905525

[2] Barrangou R, Fremaux C, Deveau H, Richards M, Boyaval P, Moineau S, Romero DA, and Horvath P (2007). CRISPR provides acquired resistance against viruses in prokaryotes. Science 315, 1709–1712. 17379808 10.1126/science.1138140

[3] Marraffini LA, and Sontheimer EJ (2008). CRISPR interference limits horizontal gene transfer in staphylococci by targeting DNA. Science 322, 1843–1845. 19095942 10.1126/science.1165771 PMC2695655

[4] Bolotin A, Quinquis B, Sorokin A, and Ehrlich SD (2005). Clustered regularly interspaced short palindrome repeats (CRISPRs) have spacers of extrachromosomal origin. Microbiology 151, 2551–2561. 16079334 10.1099/mic.0.28048-0

[5] Mojica FJM, Díez-Villaseñor C, García-Martínez J, and Soria E, (2005). Intervening sequences of regularly spaced prokaryotic repeats derive from foreign genetic elements. J. Mol. Evol 60, 174–182. 15791728 10.1007/s00239-004-0046-3

[6] Pourcel C, Salvignol G, and Vergnaud G (2005). CRISPR elements in *Yersinia pestis* acquire new repeats by preferential uptake of bacteriophage DNA, and provide additional tools for evolutionary studies. Microbiology 151, 653–663. 15758212 10.1099/mic.0.27437-0

[7] Brouns SJJ, Jore MM, Lundgren M, Westra ER, Slijkhuis RJH, Snijders APL, Dickman MJ, Makarova KS, Koonin EV, and van der Oost J (2008). Small CRISPR RNAs guide antiviral defense in prokaryotes. Science 321, 960–964. 18703739 10.1126/science.1159689 PMC5898235

[8] Hale C, Kleppe K, Terns RM, and Terns MP (2008). Prokaryotic silencing (psi)RNAs in *Pyrococcus furiosus*. RNA 14, 2572–2579. 18971321 10.1261/rna.1246808 PMC2590957

[9] Garneau JE, Dupuis M.è., Villion M, Romero DA, Barrangou R, Boyaval P, Fremaux C, Horvath P, Magadán AH, and Moineau S, (2010). The CRISPR/Cas bacterial immune system cleaves bacteriophage and plasmid DNA. Nature 468, 67–71. 21048762 10.1038/nature09523

[10] Hale CR, Zhao P, Olson S, Duff MO, Graveley BR, Wells L, Terns RM, and Terns MP (2009). RNA-guided RNA cleavage by a CRISPR RNA-Cas protein complex. Cell 139, 945–956. 19945378 10.1016/j.cell.2009.07.040 PMC2951265

[11] Jore MM, Lundgren M, van Duijn E, Bultema JB, Westra ER, Waghmare SP, Wiedenheft B, Pul U, Wurm R, Wagner R, (2011). Structural basis for CRISPR RNA-guided DNA recognition by Cascade. Nat. Struct. Mol. Biol 18, 529–536. 21460843 10.1038/nsmb.2019

[12] Eppley JM, Biller SJ, Luo E, Burger A, and DeLong EF (2022). Marine viral particles reveal an expansive repertoire of phage-parasitizing mobile elements. Proc. Natl. Acad. Sci. USA 119, e2212722119. 10.1073/pnas.2212722119. 36256808 PMC9618062

[13] Fillol-Salom A, Martínez-Rubio R, Abdulrahman RF, Chen J, Davies R, and Penadés JR, (2018). Phage-inducible chromosomal islands are ubiquitous within the bacterial universe. ISME J. 12, 2114–2128. 10.1038/s41396-018-0156-3. 29875435 PMC6092414

[14] Penades JR, and Christie GE (2015). The Phage-Inducible Chromosomal Islands: A Family of Highly Evolved Molecular Parasites. Annu. Rev. Virol 2, 181–201. 10.1146/annurev-virology-031413-085446. 26958912

[15] Lindsay JA, Ruzin A, Ross HF, Kurepina N, and Novick RP (1998). The gene for toxic shock toxin is carried by a family of mobile pathogenicity islands in Staphylococcus aureus. Mol. Microbiol 29, 527–543. 9720870 10.1046/j.1365-2958.1998.00947.x

[16] Ubeda C, Maiques E, Barry P, Matthews A, Tormo MA, Lasa I, Novick RP, and Penadés JR (2008). SaPI mutations affecting replication and transfer and enabling autonomous replication in the absence of helper phage. Mol. Microbiol 67, 493–503. 10.1111/j.1365-2958.2007.06027.x. 18086210

[17] Tormo-Mas MA, Mir I, Shrestha A, Tallent SM, Campoy S, Lasa I, Barbe J, Novick RP, Christie GE, and Penades JR (2010). Moonlighting bacteriophage proteins derepress staphylococcal pathogenicity islands. Nature 465, 779–782. 20473284 10.1038/nature09065 PMC3518041

[18] Tallent SM, Langston TB, Moran RG, and Christie GE (2007). Transducing particles of Staphylococcus aureus pathogenicity island SaPI1 are comprised of helper phage-encoded proteins. J. Bacteriol 189, 7520–7524. 10.1128/JB.00738-07. 17693489 PMC2168463

[19] Tormo MA, Ferrer MD, Maiques E, Ubeda C, Selva L, Lasa I, Calvete JJ, Novick RP, and Penadés JR (2008). Staphylococcus aureus pathogenicity island DNA is packaged in particles composed of phage proteins. J. Bacteriol 190, 2434–2440. 10.1128/JB.01349-07. 18223072 PMC2293202

[20] Ruzin A, Lindsay J, and Novick RP (2001). Molecular genetics of SaPI1–a mobile pathogenicity island in Staphylococcus aureus. Mol. Microbiol 41, 365–377. 10.1046/j.1365-2958.2001.02488.x. 11489124

[21] Viana D, Blanco J, Tormo-Más MA, Selva L, Guinane CM, Baselga R, Corpa JM, Lasa I, Novick RP, Fitzgerald JR, and Penadés JR, (2010). Adaptation of Staphylococcus aureus to ruminant and equine hosts involves SaPI-carried variants of von Willebrand factor-binding protein. Mol. Microbiol 77, 1583–1594. 10.1111/j.1365-2958.2010.07312.x. 20860091

[22] Fillol-Salom A, Rostol JT, Ojiogu AD, Chen J, Douce G, Humphrey S, and Penades JR (2022). Bacteriophages benefit from mobilizing pathogenicity islands encoding immune systems against competitors. Cell 185, 3248–3262.e3220. 10.1016/j.cell.2022.07.014. 35985290

[23] Ran FA, Cong L, Yan WX, Scott DA, Gootenberg JS, Kriz AJ, Zetsche B, Shalem O, Wu X, Makarova KS, (2015). In vivo genome editing using Staphylococcus aureus Cas9. Nature 520, 186–191. 10.1038/nature14299. 25830891 PMC4393360

[24] Samai P, Pyenson N, Jiang W, Goldberg GW, Hatoum-Aslan A, and Marraffini LA (2015). Co-transcriptional DNA and RNA Cleavage during Type III CRISPR-Cas Immunity. Cell 161, 1164–1174. 10.1016/j.cell.2015.04.027. 25959775 PMC4594840

[25] Kazlauskiene M, Tamulaitis G, Kostiuk G, Venclovas ć., and Siksnys V (2016). Spatiotemporal Control of Type III-A CRISPR-Cas Immunity: Coupling DNA Degradation with the Target RNA Recognition. Mol. Cell 62, 295–306. 10.1016/j.molcel.2016.03.024. 27105119

[26] Kazlauskiene M, Kostiuk G, Venclovas ć., Tamulaitis G, and Siksnys V (2017). A cyclic oligonucleotide signaling pathway in type III CRISPR-Cas systems. Science 357, 605–609. 10.1126/science.aao0100. 28663439

[27] Niewoehner O, Garcia-Doval C, Rostøl JT, Berk C, Schwede F, Bigler L, Hall J, Marraffini LA, and Jinek M (2017). Type III CRISPR-Cas systems produce cyclic oligoadenylate second messengers. Nature 548, 543–548. 10.1038/nature23467. 28722012

[28] Jiang W, Samai P, and Marraffini LA (2016). Degradation of phage transcripts by CRISPR-associated RNases enables type III CRISPR-Cas immunity. Cell 164, 710–721. 10.1016/j.cell.2015.12.053. 26853474 PMC4752873

[29] Rostol JT, and Marraffini LA (2019). Non-specific degradation of transcripts promotes plasmid clearance during type III-A CRISPR-Cas immunity. Nat. Microbiol 4, 656–662. 10.1038/s41564-018-0353-x. 30692669 PMC6430669

[30] Miguel-Romero L, Alqasmi M, Bacarizo J, Tan JA, Cogdell RJ, Chen J, Byron O, Christie GE, Marina A, and Penadés JR (2022). Non-canonical Staphylococcus aureus pathogenicity island repression. Nucleic Acids Res. 50, 11109–11127. 10.1093/nar/gkac855. 36200825 PMC9638917

[31] Spilman MS, Dearborn AD, Chang JR, Damle PK, Christie GE, and Dokland T (2011). A conformational switch involved in maturation of Staphylococcus aureus bacteriophage 80alpha capsids. J. Mol. Biol 405, 863–876. 10.1016/j.jmb.2010.11.047. 21129380 PMC3017672

[32] Dearborn AD, Spilman MS, Damle PK, Chang JR, Monroe EB, Saad JS, Christie GE, and Dokland T (2011). The Staphylococcus aureus pathogenicity island 1 protein gp6 functions as an internal scaffold during capsid size determination. J. Mol. Biol 412, 710–722. 10.1016/j.jmb.2011.07.036. 21821042 PMC3175317

[33] Ubeda C, Olivarez NP, Barry P, Wang H, Kong X, Matthews A, Tallent SM, Christie GE, and Novick RP (2009). Specificity of staphylococcal phage and SaPI DNA packaging as revealed by integrase and terminase mutations. Mol. Microbiol 72, 98–108. 19347993 10.1111/j.1365-2958.2009.06634.x PMC3885990

[34] Christie GE, Matthews AM, King DG, Lane KD, Olivarez NP, Tallent SM, Gill SR, and Novick RP (2010). The complete genomes of Staphylococcus aureus bacteriophages 80 and 80alpha–implications for the specificity of SaPI mobilization. Virology 407, 381–390. 10.1016/j.virol.2010.08.036. 20869739 PMC2952651

[35] Cao L, Gao CH, Zhu J, Zhao L, Wu Q, Li M, and Sun B (2016). Identification and functional study of type III-A CRISPR-Cas systems in clinical isolates of Staphylococcus aureus. Int. J. Med. Microbiol 306, 686–696. 10.1016/j.ijmm.2016.08.005. 27600408

[36] Martinez-Rubio R, Quiles-Puchalt N, Marti M, Humphrey S, Ram G, Smyth D, Chen J, Novick RP, and Penades JR (2017). Phage-inducible islands in the Gram-positive cocci. ISME J. 11, 1029–1042. 10.1038/ismej.2016.163. 27959343 PMC5363835

[37] Pourcel C, Touchon M, Villeriot N, Vernadet JP, Couvin D, Toffano-Nioche C, and Vergnaud G (2020). CRISPRCasdb a successor of CRISPRdb containing CRISPR arrays and cas genes from complete genome sequences, and tools to download and query lists of repeats and spacers. Nucleic Acids Res. 48, D535–D544. 10.1093/nar/gkz915. 31624845 PMC7145573

[38] de Sousa JAM, Fillol-Salom A, Penades JR, and Rocha EPC (2023). Identification and characterization of thousands of bacteriophage satellites across bacteria. Nucleic Acids Res. 51, 2759–2777. 10.1093/nar/gkad123. 36869669 PMC10085698

[39] Kinnevey PM, Shore AC, Brennan GI, Sullivan DJ, Ehricht R, Monecke S, Slickers P, and Coleman DC (2013). Emergence of sequence type 779 methicillin-resistant Staphylococcus aureus harboring a novel pseudo staphylococcal cassette chromosome mec (SCCmec)-SCC-SCCCRISPR composite element in Irish hospitals. Antimicrob. Agents Chemother 57, 524–531. 10.1128/AAC.01689-12. 23147725 PMC3535981

[40] Gill SR, Fouts DE, Archer GL, Mongodin EF, Deboy RT, Ravel J, Paulsen IT, Kolonay JF, Brinkac L, Beanan M, (2005). Insights on evolution of virulence and resistance from the complete genome analysis of an early methicillin-resistant *Staphylococcus aureus* strain and a biofilm-producing methicillin-resistant *Staphylococcus epidermidis* strain. J. Bacteriol 187, 2426–2438. 15774886 10.1128/JB.187.7.2426-2438.2005 PMC1065214

[41] Bae T, Baba T, Hiramatsu K, and Schneewind O (2006). Prophages of *Staphylococcus aureus* Newman and their contribution to virulence. Mol. Microbiol 62, 1035–1047. 10.1111/j.1365-2958.2006.05441.x. 17078814

[42] Banh DV, Roberts CG, Morales-Amador A, Berryhill BA, Chaudhry W, Levin BR, Brady SF, and Marraffini LA (2023). Bacterial cGAS senses a viral RNA to initiate immunity. Nature 623, 1001–1008. 10.1038/s41586-023-06743-9. 37968393 PMC10686824

[43] Ibarra-Chavez R, Brady A, Chen J, Penades JR, and Haag AF (2022). Phage-inducible chromosomal islands promote genetic variability by blocking phage reproduction and protecting transductants from phage lysis. PLoS Genet. 18, e1010146. 10.1371/journal.pgen.1010146. 35344558 PMC8989297

[44] Frigols B, Quiles-Puchalt N, Mir-Sanchis I, Donderis J, Elena SF, Buckling A, Novick RP, Marina A, and Penades JR (2015). Virus satellites drive viral evolution and ecology. PLoS Genet. 11, e1005609. 10.1371/journal.pgen.1005609. 26495848 PMC4619825

[45] Buckling A, and Rainey PB (2002). Antagonistic coevolution between a bacterium and a bacteriophage. Proc. Biol. Sci 269, 931–936. 10.1098/rspb.2001.1945. 12028776 PMC1690980

[46] Horinouchi S, and Weisblum B (1982). Nucleotide sequence and functional map of pC194, a plasmid that specifies inducible chloramphenicol resistance. J. Bacteriol 150, 815–825. 6950931 10.1128/jb.150.2.815-825.1982 PMC216434

[47] Heler R, Samai P, Modell JW, Weiner C, Goldberg GW, Bikard D, and Marraffini LA (2015). Cas9 specifies functional viral targets during CRISPR-Cas adaptation. Nature 519, 199–202. 10.1038/nature14245. 25707807 PMC4385744

[48] Jakhanwal S, Cress BF, Maguin P, Lobba MJ, Marraffini LA, and Doudna JA (2021). A CRISPR-Cas9-integrase complex generates precise DNA fragments for genome integration. Nucleic Acids Res. 49, 3546–3556. 10.1093/nar/gkab123. 33693715 PMC8034620

[49] Nunez JK, Kranzusch PJ, Noeske J, Wright AV, Davies CW, and Doudna JA (2014). Cas1-Cas2 complex formation mediates spacer acquisition during CRISPR-Cas adaptive immunity. Nat. Struct. Mol. Biol 21, 528–534. 10.1038/nsmb.2820. 24793649 PMC4075942

[50] Maguin P, Varble A, Modell JW, and Marraffini LA (2022). Cleavage of viral DNA by restriction endonucleases stimulates the type II CRISPR-Cas immune response. Mol. Cell 82, 907–919.e7. 10.1016/j.molcel.2022.01.012. 35134339 PMC8900293

[51] Hynes AP, Villion M, and Moineau S (2014). Adaptation in bacterial CRISPR-Cas immunity can be driven by defective phages. Nat. Commun 5, 4399. 10.1038/ncomms5399. 25056268

[52] Nussenzweig PM, McGinn J, and Marraffini LA (2019). Cas9 Cleavage of Viral Genomes Primes the Acquisition of New Immunological Memories. Cell Host Microbe 26, 515–526.e6. 10.1016/j.chom.2019.09.002. 31585845 PMC7558852

[53] Bento JC, Lane KD, Read EK, Cerca N, and Christie GE (2014). Sequence determinants for DNA packaging specificity in the *S. aureus* pathogenicity island SaPI1. Plasmid 71, 8–15. 10.1016/j.plasmid.2013.12.001. 24365721 PMC3966571

[54] van Houte S, Ekroth AKE, Broniewski JM, Chabas H, Ashby B, Bondy-Denomy J, Gandon S, Boots M, Paterson S, Buckling A, and Westra ER (2016). The diversity-generating benefits of a prokaryotic adaptive immune system. Nature 532, 385–388. 10.1038/nature17436. 27074511 PMC4935084

[55] Deveau H, Barrangou R, Garneau JE, Labonté J, Fremaux C, Boyaval P, Romero DA, Horvath P, and Moineau S (2008). Phage response to CRISPR-encoded resistance in *Streptococcus thermophilus*. J. Bacteriol 190, 1390–1400. 18065545 10.1128/JB.01412-07 PMC2238228

[56] Aviram N, Thornal AN, Zeevi D, and Marraffini LA (2022). Different modes of spacer acquisition by the *Staphylococcus epidermidis* type III-A CRISPR-Cas system. Nucleic Acids Res. 50, 1661–1672. 10.1093/nar/gkab1299. 35048966 PMC8860600

[57] Zhang X, Garrett S, Graveley BR, and Terns MP (2022). Unique properties of spacer acquisition by the type III-A CRISPR-Cas system. Nucleic Acids Res. 50, 1562–1582. 10.1093/nar/gkab1193. 34893878 PMC8860593

[58] Artamonova D, Karneyeva K, Medvedeva S, Klimuk E, Kolesnik M, Yasinskaya A, Samolygo A, and Severinov K (2020). Spacer acquisition by Type III CRISPR-Cas system during bacteriophage infection of Thermus thermophilus. Nucleic Acids Res. 48, 9787–9803. 10.1093/nar/gkaa685. 32821943 PMC7515739

[59] Goldberg GW, Jiang W, Bikard D, and Marraffini LA (2014). Conditional tolerance of temperate phages via transcription-dependent CRISPR-Cas targeting. Nature 514, 633–637. 10.1038/nature13637. 25174707 PMC4214910

[60] Rostol JT, Xie W, Kuryavyi V, Maguin P, Kao K, Froom R, Patel DJ, and Marraffini LA (2021). The Card1 nuclease provides defence during type III CRISPR immunity. Nature 590, 624–629. 10.1038/s41586-021-03206-x. 33461211 PMC7906951

[61] Pyenson NC, Gayvert K, Varble A, Elemento O, and Marraffini LA (2017). Broad Targeting Specificity during Bacterial Type III CRISPR-Cas Immunity Constrains Viral Escape. Cell Host Microbe 22, 343–353.e3. 10.1016/j.chom.2017.07.016. 28826839 PMC5599366

[62] Lowy FD (1998). *Staphylococcus aureus* infections. N. Engl. J. Med 339, 520–532. 9709046 10.1056/NEJM199808203390806

[63] Murphy K, and Weaver C (2017). Janeway’s Immunobiology, 9th Edition (Garland Science, Taylor & Francis Group and Limited Liability Company), pp. 473–486.

[64] Nunez JK, Lee AS, Engelman A, and Doudna JA (2015). Integrase-mediated spacer acquisition during CRISPR-Cas adaptive immunity. Nature 519, 193–198. 10.1038/nature14237. 25707795 PMC4359072

[65] Yosef I, Goren MG, and Qimron U (2012). Proteins and DNA elements essential for the CRISPR adaptation process in *Escherichia coli*. Nucleic Acids Res. 40, 5569–5576. 10.1093/nar/gks216. 22402487 PMC3384332

[66] Dimitriu T, Kurilovich E, Lapinska U, Severinov K, Pagliara S, Szczelkun MD, and Westra ER (2022). Bacteriostatic antibiotics promote CRISPR-Cas adaptive immunity by enabling increased spacer acquisition. Cell Host Microbe 30, 31–40.e35. 10.1016/j.chom.2021.11.014. 34932986

[67] Boyd CM, Subramanian S, Dunham DT, Parent KN, and Seed KD (2023). A Vibrio cholerae viral satellite maximizes its spread and inhibits phage by remodeling hijacked phage coat proteins into small capsids. bioRxiv 337, 816. 10.1101/2023.03.01.530633. PMC10945586 38206122

[68] Jinek M, Chylinski K, Fonfara I, Hauer M, Doudna JA, and Charpentier E (2012). A programmable dual-RNA-guided DNA endonuclease in adaptive bacterial immunity. Science 337, 816–821. 10.1126/science.1225829. 22745249 PMC6286148

[69] Meeske AJ, Nakandakari-Higa S, and Marraffini LA (2019). Cas13-induced cellular dormancy prevents the rise of CRISPR-resistant bacteriophage. Nature 570, 241–245. 10.1038/s41586-019-1257-5. 31142834 PMC6570424

[70] Seed KD, Lazinski DW, Calderwood SB, and Camilli A (2013). A bacteriophage encodes its own CRISPR/Cas adaptive response to evade host innate immunity. Nature 494, 489–491. 10.1038/nature11927. 23446421 PMC3587790

[71] Jiang W, Maniv I, Arain F, Wang Y, Levin BR, and Marraffini LA (2013). Dealing with the evolutionary downside of CRISPR immunity: bacteria and beneficial plasmids. PLoS Genet. 9, e1003844. 10.1371/journal.pgen.1003844. 24086164 PMC3784566

[72] Palmer KL, and Gilmore MS (2010). Multidrug-resistant enterococci lack CRISPR-cas. mBio 1, e00227–10. 21060735 10.1128/mBio.00227-10 PMC2975353

[73] Novick RP, Christie GE, and Penadés JR (2010). The phage-related chromosomal islands of Gram-positive bacteria. Nat. Rev. Microbiol 8, 541–551. 10.1038/nrmicro2393. 20634809 PMC3522866

[74] Pursey E, Dimitriu T, Paganelli FL, Westra ER, and van Houte S (2022). CRISPR-Cas is associated with fewer antibiotic resistance genes in bacterial pathogens. Philos. Trans. R. Soc. Lond. B Biol. Sci 377, 20200464. 10.1098/rstb.2020.0464. 34839714 PMC8628084

[75] Kreiswirth BN, Löfdahl S, Betley MJ, O’Reilly M, Schlievert PM, Bergdoll MS, and Novick RP (1983). The toxic shock syndrome exotoxin structural gene is not detectably transmitted by a prophage. Nature 305, 709–712. 6226876 10.1038/305709a0

[76] Schneewind O, Model P, and Fischetti VA (1992). Sorting of protein A to the staphylococcal cell wall. Cell 70, 267–281. 1638631 10.1016/0092-8674(92)90101-h

[77] Damle PK, Wall EA, Spilman MS, Dearborn AD, Ram G, Novick RP, Dokland T, and Christie GE (2012). The roles of SaPI1 proteins gp7 (CpmA) and gp6 (CpmB) in capsid size determination and helper phage interference. Virology 432, 277–282. 10.1016/j.virol.2012.05.026. 22709958 PMC3423473

[78] Gibson DG, Young L, Chuang RY, Venter JC, Hutchison CA 3rd, and Smith HO (2009). Enzymatic assembly of DNA molecules up to several hundred kilobases. Nat. Methods 6, 343–345. 10.1038/nmeth.1318. 19363495

[79] Schneewind O, and Missiakas D (2014). Genetic manipulation of Staphylococcus aureus. Curr. Protoc. Microbiol 32, Unit 9C 3. 10.1002/9780471729259.mc09c03s32. PMC6249557 24510849

[80] Jakociune D, and Moodley A (2018). A Rapid Bacteriophage DNA Extraction Method. Methods Protoc. 1, 27. 10.3390/mps1030027.80. 31164569 PMC6481073

[81] Galaxy C (2022). The Galaxy platform for accessible, reproducible and collaborative biomedical analyses: 2022 update. Nucleic Acids Res. 50, W345–W351. 10.1093/nar/gkac247. 35446428 PMC9252830

