# Supplementary material for: Phage induction of Staphylococcus aureus pathogenicity islands promotes the CRISPR-Cas adaptive immune response
Source: Cell Rep. Author manuscript; Available in PMC 2026 Feb 24. (PMC12931967; doi:10.1016/j.celrep.2025.116776)
Supplement: 1 [file NIHMS2142883-supplement-1.pdf]

Cell Reports, Volume *45*

Supplemental information

Phage induction of *Staphylococcus aureus* pathogenicity islands promotes the CRISPR-Cas adaptive immune response

Dalton V. Banh, Gregory W. Goldberg, and Luciano A. Marraffini

SUPPLEMENTARY FIGURES AND LEGENDS

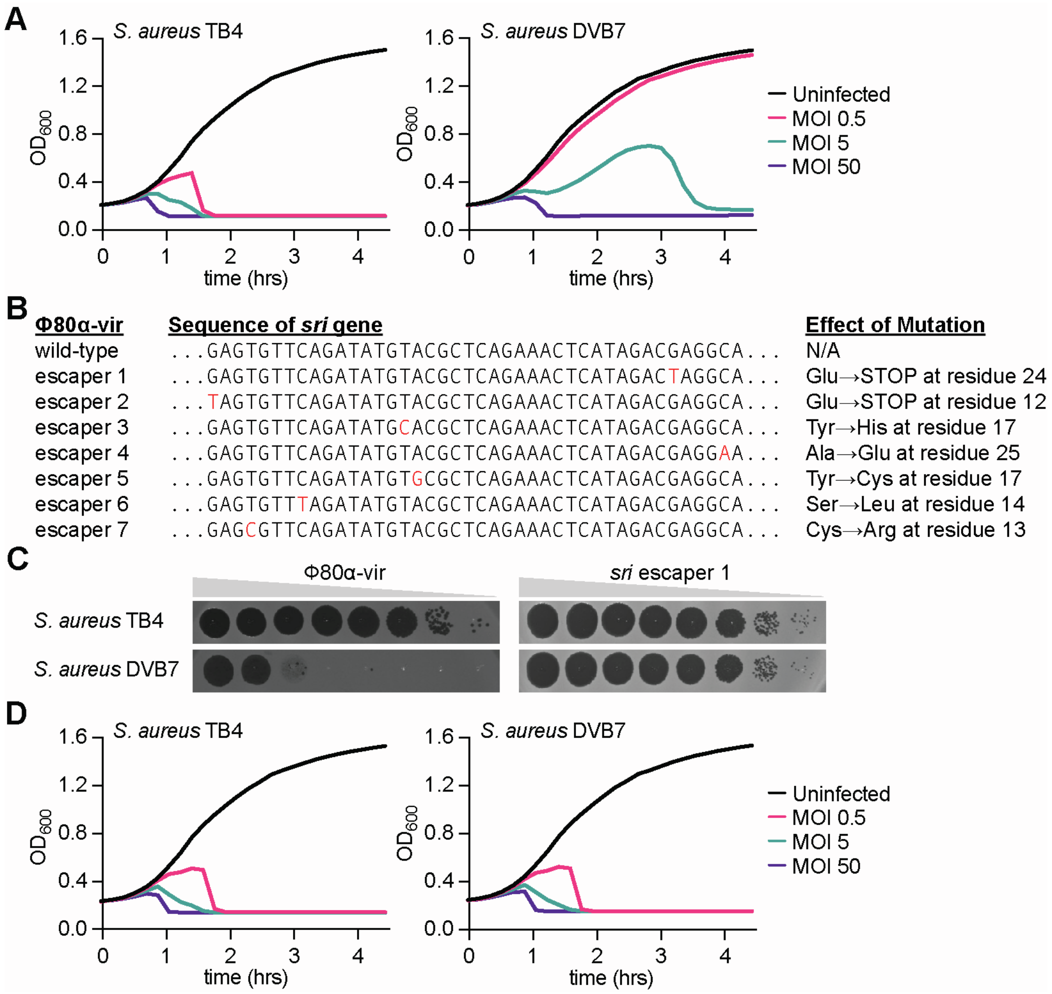

Figure S1. Φ80α-vir phages harboring mutations in *sri* escape SaPI1-mediated interference. Related to Fig. 1.

(A) Growth of *S. aureus* TB4 (SaPI-) or DVB7 (SaPI+) strains, measured by optical density at 600nm (OD_{600}), after infection with Φ80α-vir at different multiplicities of infection (MOI). The mean for 3 biological replicates is shown.

(B) Sequences of SaPI1-escaper phages, showing mutated nucleotides in red, as well as their effects on the amino acid sequence of the Sri protein.

(C) Detection of plaque formation after seeding 10-fold dilutions of Φ80α-vir, or the SaPI1-escaper #1, on lawns of TB4 (SaPI-) or DVB7 (SaPI+) staphylococci.

(D) Same as **(A)** but performing infections with Φ80α-vir SaPI1-escaper #1.

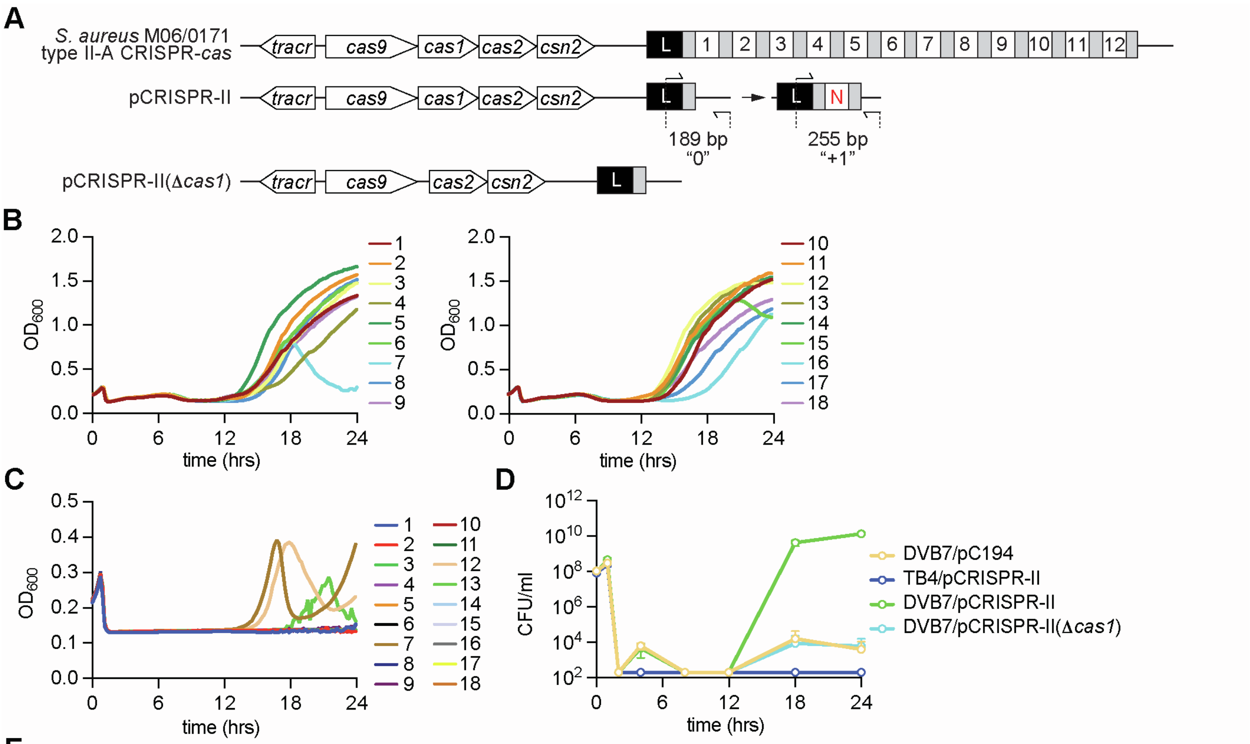

E

colony from culture #	spacer sequence (5' to 3')	PAM (5'-NNGRRT-3')	location on Φ80α-vir
1	TTTGTTCGTAACATTAGTAGGTGCTATGG	CAGAGT	gp01
2	TCGATTATAGTTTGCTGTACGAAAGAATGG	CAGAGT	gp07
3	GATGGTACAATTGCACTCGTTGTATTAGA	TGGAGT	gp06
4	ACTTTTCTTTCAATATCGTTGATAGTGGTA	TTGAAT	gp68
5	AAGGATTTAAAGATATGATTCGAGAAGCTA	AAGAGT	gp04
6	AAGCAATCAAGAAAACAGTGACAGAAACTA	TTGAGT	gp15
7	TAAAACTGTTCATATTTCTTAACACCTCAT	AAGAAT	gp06
8	GTTTTTACAGAGGTTTAATTTATATTCACG	GTGGGT	gp03
9	TTTTAACTAGTGGTAATCAACCATTCACTT	ATGAGT	gp05
10	GATTTTAAGATTTCATCAACATAATCTGCT	TTGGGT	gp08
11	GATTAGATTTGTCAATTTATGGTAAAGCTT	ATGAGT	gp42
12	TAACAACTCTATGTTGTTAAGATATATGTA	TCGAGT	gp72
13	CAGTGGGTGCTTTACTCAGAACACATGGGC	AAGAGT	gp08
14	TTATGACATTTAAAAACAATCATAATTTCA	ATGAAT	gp02
15	ACGTATGTGAAGATTATAGTGCGAATAAAA	ATGAAT	gp67
16	GATAATGCTGAATCAGATACTGCTAACTAT	ATGAGT	gp42
17	AAAATATTCTAATTTATTCATGCAATATAT	ATGGGT	gp73
18	TATTCTTTGAAAAAGCATTCTTTGTAGTAC	ATGAAT	gp13

Figure S2. SaPI1 induction triggers Type II-A CRISPR spacer acquisition. Related to Fig. 1.

(A) Genomic architecture of the type II-A CRISPR-*cas* locus from *S. aureus* M06/0171. CRISPR repeats are denoted as grey boxes, the leader (L) sequence as a black box preceding the first repeat, and spacers as white boxes with different numbers. To construct the pCRISPR-II plasmid, this locus was cloned into the staphylococcal vector pC194, and the CRISPR array was reduced to a single repeat, without any pre-existing spacer. Spacer acquisition was measured via amplification of this "minimal CRISPR". The primers used are shown as arrows, indicating the size of the PCR products before and after the acquisition of a new spacer (red "N"), denoted as "0" and "+1,"

respectively, in Figures 1E-F and 2C-E. pCRISPR-II(Δ*cas1*) was generated by deletion of *cas1*.

(B) Growth of the 18 different *S. aureus* DVB7/pCRISPR-II cultures shown in Figure 1C, measured by optical density at 600nm (OD_{600}) after infection with Φ80α-vir at MOI 10.

(C) Growth of the 18 different *S. aureus* TB4/pCRISPR-II cultures shown in Figure 1C, measured by optical density at 600nm (OD_{600}) after infection with Φ80α-vir at MOI 10.

(D) Growth of different *S. aureus* strains measured by the CFU/ml present in aliquots of cultures taken at 0, 1, 2, 4, 8, 12, 16 and 24 hours after infection with Φ80α-vir at MOI 10. The mean ± S.D. for 3 biological replicates is shown.

(E) Sequences of the spacers present in colonies obtained after plating the cultures shown in **(B)**, showing both their corresponding PAM sequence and gene of origin in the Φ80α-vir genome.

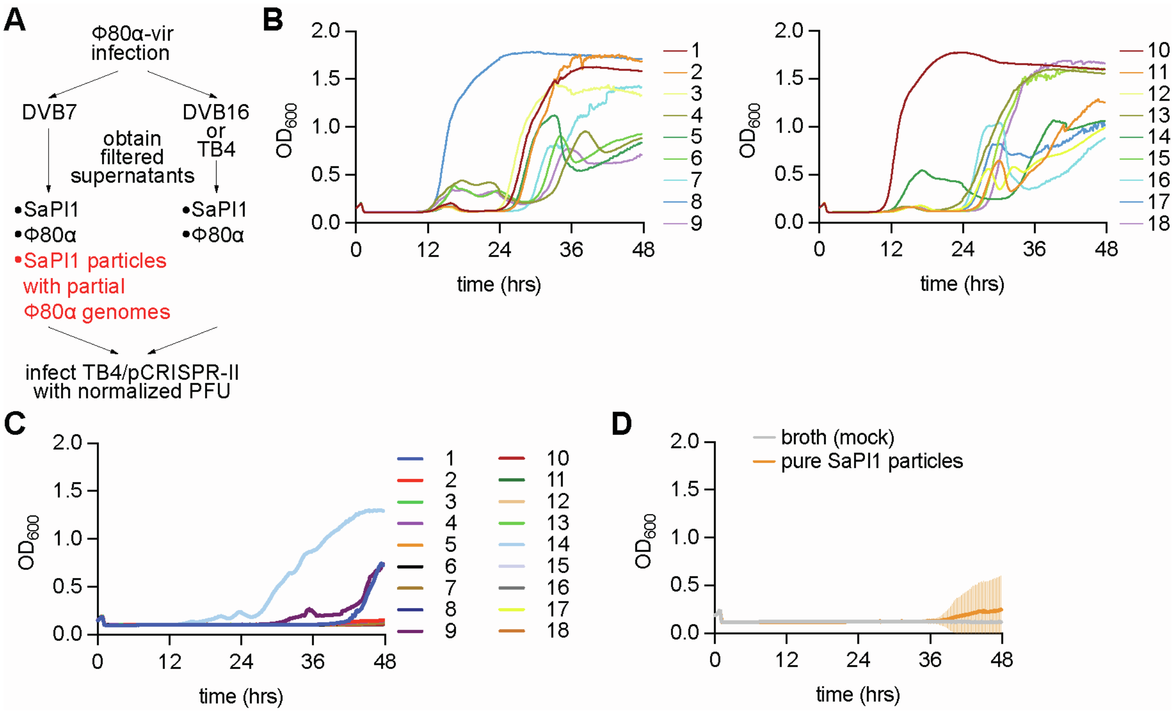

Figure S3. SaPI1 particles containing partial Φ80α-vir genomes enhance Type II-A CRISPR spacer acquisition. Related to Fig. 2.

(A) Schematic for the generation of infection lysates containing a mixture of Φ80α-vir and SaPI1, and/or SaPI1-modified particles containing partial phage genomes used to infect DVB7/pCRISPR-II cultures shown in Figure 2B.

(B) Growth of the 18 different *S. aureus* TB4/pCRISPR-II cultures shown in Figure 2B, measured by optical density at 600nm (OD_{600}) after infection with a *S. aureus* DVB7 (SaPI+) Φ80α-vir lysate.

(C) Same as **(B)** but performing infections with a *S. aureus* DVB16 Φ80α-vir lysate.

(D) Growth of *S. aureus* TB4/pCRISPR-II cultures, measured by optical density at 600nm (OD_{600}) after infection with a *S. aureus* TB4 (SaPI-) Φ80α-vir lysate supplemented with pure SaPI1 particles. These particles were obtained after induction of *S. aureus* ST16, which is a strain of RN4220 harboring SaPI1 *tst::tetM* and lysogenized with Φ80α containing a *terS* deletion (Φ80α Δ*terS*), a mutation that prevents the packaging of the viral DNA into SaPI1 particles. As a control, culture broth was supplemented instead of SaPI1 particles. The mean ± S.D. for 12 biological replicates is shown.

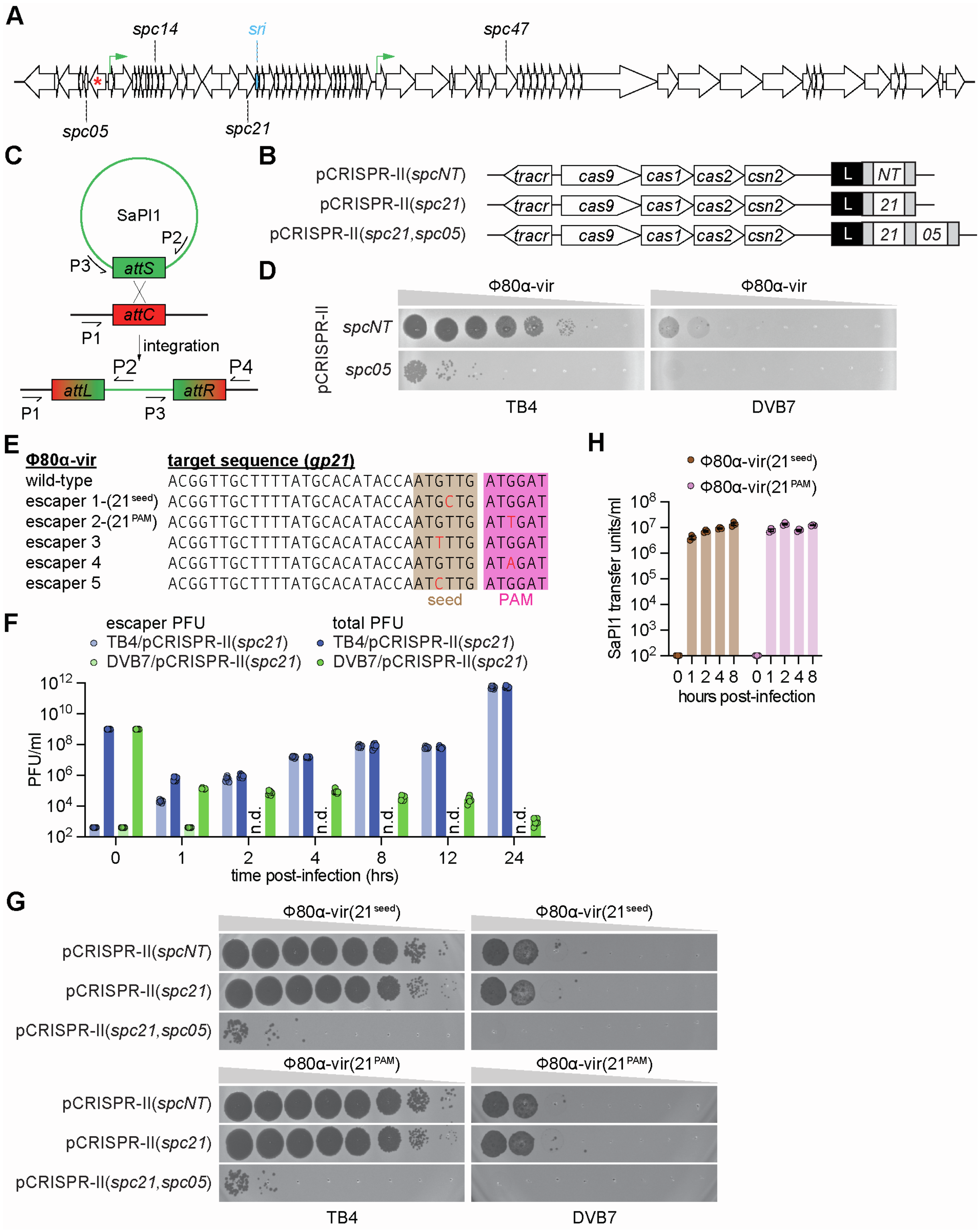

Figure S4. Isolation and characterization of SaPI1 induction by Φ80α-vir escaper phages that evade pCRISPR-II(*spc21*) targeting. Related to Fig. 3.

(A) Genomic architecture of the Φ80α-vir genome showing the location of the type II-A and III-A CRISPR spacers used in this study. Each spacer generates a crRNA, complementary to the top or bottom strand of the viral DNA, and is depicted on either the top or bottom, respectively. The *sri* gene (*gp22*) responsible for SaPI1 induction is shown in blue. The gene mutated in Φ80α to obtain a virulent variant (*gp06*) is marked with a red asterisk. The green arrows indicate the two promoters that drive early and late gene expression during the viral lytic cycle.

(B) pCRISPR-II plasmids used in this study, harboring a non-targeting spacer (*spcNT*), *spc21*, or both *spc21* and *spc05*.

(C) Multiplex PCR assay to measure the integrated and/or excised forms of SaPI1. Primers 2/3 flank the SaPI1 attachment site, *attS*; amplification with these primers reflect the presence of the excised form in the culture. Primers 1/2 and 3/4 flank the left (*attL*) and right (*attR*) attachment sites, respectively, in the staphylococcal chromosome; amplification with these primers reflect the presence of the integrated SaPI1 form.

(D) Detection of plaque formation after seeding 10-fold dilutions of Φ80α-vir on lawns of TB4 (SaPI-) or DVB7 (SaPI+) staphylococci harboring pCRISPR-II plasmids programmed with *spc05*.

(E) Sequences of the protospacer, seed, and PAM sequences of phages that escape pCRISPR-II(*spc21*) targeting, with mutated nucleotides shown in red. Escapers #1 and #2 were renamed Φ80α-vir(21^{seed}) and Φ80α-vir(21^{PAM}), respectively.

(F) Enumeration of plaque-forming units (PFU) per milliliter of filtered supernatants taken at different times after infection of *S. aureus* TB4 (SaPI-) or DVB7 (SaPI+) carrying pCRISPR-II(*spc21*) with Φ80α-vir at MOI 10, plated on lawns of *S. aureus* TB4 (SaPI-) or TB4/pCRISPR-II(*spc21*) to determine total or escaper PFU, respectively. Mean $\pm$ S.E.M. of 6 biological replicates is shown. The escaper/total PFU ratio was calculated to obtain the data of Figure 3E.

(G) Detection of plaque formation after seeding 10-fold dilutions of Φ80α-vir(21^{seed}) or Φ80α-vir(21^{PAM}) on lawns of TB4 (SaPI-) or DVB7 (SaPI+) staphylococci harboring pCRISPR-II plasmids programmed with different spacer sequences. **(H)** Enumeration of SaPI1 transfer units per milliliter of filtered supernatants taken at increasing times after infection of *S. aureus* DVB7/pCRISPR-II(*spc21*) cultures with Φ80α-vir(21^{seed}) or Φ80α-vir(21^{PAM}). The mean $\pm$ S.E.M. of 3 biological replicates is shown.

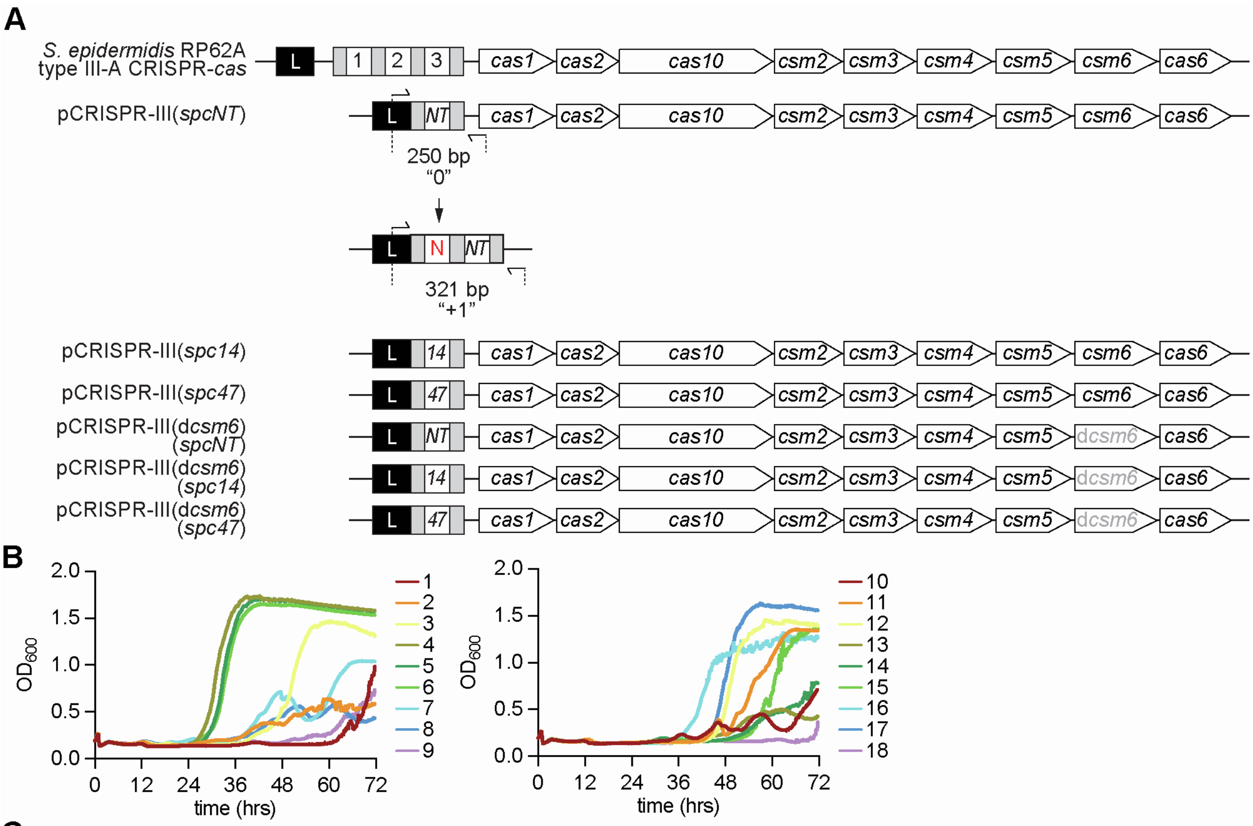

C

colony from culture #	spacer sequence (5' to 3')	location on Φ80α-vir
3	TATTCCTCTTGTAATCAGGTGTTTGTTTAATTATA	gp03
4	CCTTTTTGTGTCCATCTAGTTTGTAAAACTGTGT	gp08
5	TCTTATACTCAGTCAACACTGTAATGATGTAGTCTG	gp08
6	CCAAGTCGCAACTGCCATTGTGATGAGGAAGGTT	gp10
8	GAAGATGCTCCTGAAAACGTATCTTACGAAATTAT	gp11
9	CATGTGTTCTGAGTAAAGCACCCACTGTTTATTTA	gp08
10	TGTCTGATTCTGTGTAACTCTTGCCCATGTGTTCT	gp08
11	CAAATAAGGTACAAAATAATTTTCTGTGTTAATAT	gp16
12	TAAACTCTATCTGAAATAGTTTTAATTCTAACCTC	gp34
15	TGATATCTGCTAATAAATAATGTTCCAAATTCCCA	gp09
16	CCAACTTATATTAGAATCATGTATAAAATCTTTGT	gp20
17	TCGGTTTGTAATAAATAGATTTTCATCTTTATGC	gp15

Figure S5. SaPI1 induction enhances Type III-A CRISPR spacer acquisition. Related to Fig. 4.

(A) Genomic architecture of the type III-A CRISPR-*cas* locus from *S. epidermidis* RP62A. CRISPR repeats are denoted as grey boxes, the leader (L) sequence as a black box preceding the first repeat, and spacers as white boxes with different numbers. To construct the pCRISPR-III plasmids, this locus was cloned into the staphylococcal vector pC194, and the CRISPR array was reduced to a single repeat-spacer-repeat sequence, with different plasmids harboring different spacers. Spacer acquisition was performed using pCRISPR-III(*spcNT*) and measured via PCR. The primers used are shown as arrows, indicating the size of the PCR products before and after the acquisition of a new spacer (red "N"), noted as "0" and "+1," respectively, in Figures 4B-

C. pCRISPR-III(d*csm6*) was generated by the mutation of residues in the active site of Csm6, shown in grey.

(B) Growth of the 18 different *S. aureus* DVB7/pCRISPR-II cultures shown in Figure 4B, measured by optical density at 600nm (OD_{600}) after infection with Φ80α-vir at MOI 10.

(C) Sequences of the spacers present in colonies obtained after plating the cultures shown in **(B)**, showing their gene of origin in the Φ80α-vir genome.

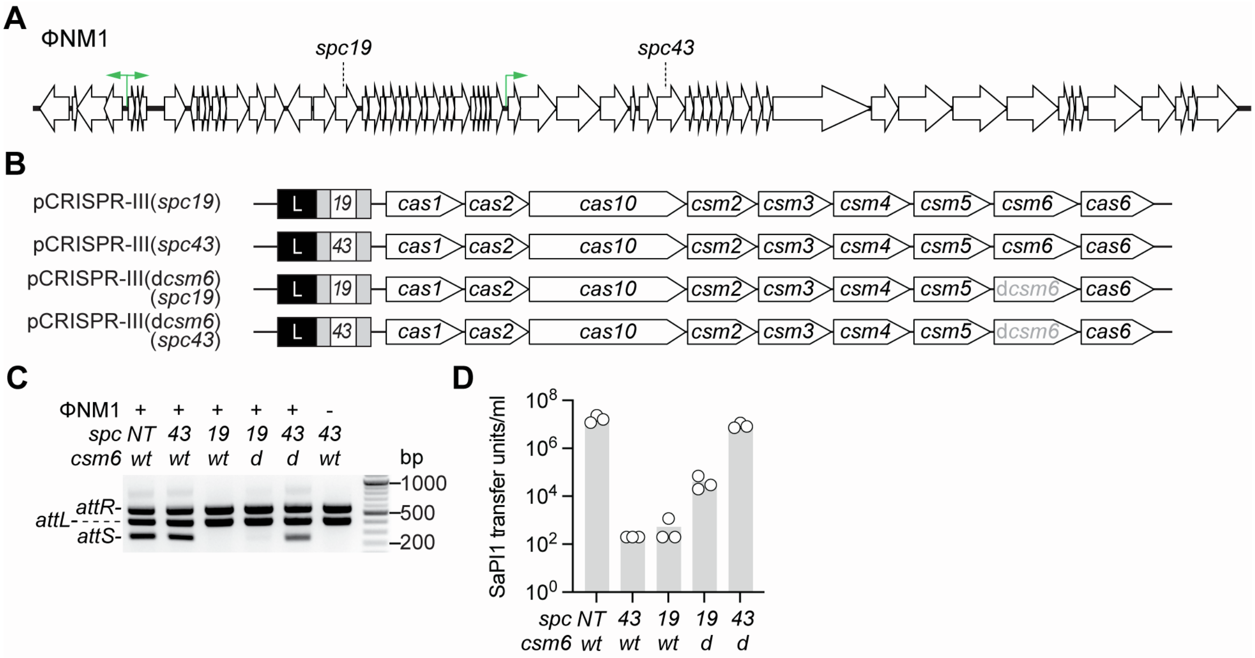

Figure S6. SaPI1 induction by the ΦNM1 prophage. Related to Fig. 5.

(A) Genomic architecture of the ΦNM1 genome showing the location of the III-A CRISPR spacers used in this study. Each spacer generates a crRNA, complementary to the top strand of the viral DNA, and therefore complementary to the viral transcripts. The green arrows indicate the two promoters that drive early and late gene expression.

(B) pCRISPR-III plasmids used in this study, harboring a non-targeting spacer (*spcNT*), *spc14* or *spc43*, in either a wild-type or catalytically dead ("d") *csm6* genetic background.

(C) Detection of the integrated (*attL*) or excised (*attS*) forms of SaPI1 via multiplex PCR 4 hours after induction by mitomycin C. Amplification products were obtained using template DNA extracted from *S. aureus* DVB3 (SaPI+ and ΦNM1 prophage) or DVB7 (SaPI+ and no prophage) cultures harboring pCRISPR-III or pCRISPR-III(d*csm6*) plasmids programmed with different spacer sequences and induced with mitomycin C, and were separated through agarose gel electrophoresis.

(D) Enumeration of SaPI1 transfer units per milliliter of filtered supernatants taken at 4 hours after induction by mitomycin C of *S. aureus* DVB3 (SaPI+ and ΦNM1 prophage) cultures harboring pCRISPR-III or pCRISPR-III(d*csm6*) plasmids programmed with different spacer sequences. The mean ± S.E.M. of 3 biological replicates is shown.

SUPPLEMENTARY TABLES

Table S1. Co-occurrence of type II-A and type III-A CRISPR-Cas loci with PICIs in staphylococci. Related to Fig. 1.

Staphylococcus Species	Strain	Type II-A CRISPR-Cas	Type III-A CRISPR-Cas	Putative PICI?	Citation (PMID)
Staphylococcus agnetis	1379	Yes	No	No	32245765
Staphylococcus agnetis	908	Yes	No	No	26606420
Staphylococcus agnetis	12B	Yes	No	Yes	32245765
Staphylococcus argenteus	1710042798	No	Yes	No	35372120
Staphylococcus argenteus	1801221	No	Yes	No	35372120
Staphylococcus argenteus	TWCC 58113	No	Yes	Yes	31023804
Staphylococcus argenteus	MSHR1132	No	Yes	No	21813488
Staphylococcus argenteus	XNO106	No	Yes	Yes	30013523
Staphylococcus argenteus	XNO62	No	Yes	Yes	30013523
Staphylococcus aureus	M06/0171	Yes	No	Yes	23147725
Staphylococcus aureus	HL25274	No	Yes	Yes	N/A
Staphylococcus aureus	110900	No	Yes	Yes	33033131
Staphylococcus aureus	AR_0470	No	Yes	Yes	29118174
Staphylococcus aureus	AR_0473	No	Yes	Yes	29118174
Staphylococcus aureus	ER03868.3	No	Yes	Yes	31578260
Staphylococcus aureus	KUH140087	No	Yes	Yes	31753944
Staphylococcus aureus	WH39	No	Yes	Yes	35170993
Staphylococcus aureus	08BA02176	No	Yes	Yes	23144384
Staphylococcus aureus	JS395	No	Yes	Yes	28088766
Staphylococcus chromogenes	17A	Yes	No	Yes	31753946
Staphylococcus coagulans	1031336	Yes	No	Yes	35366350
Staphylococcus delphini	NCTC12225	Yes	Yes	No	31023808
Staphylococcus epidermidis	FDAARGOS_153	No	Yes	No	31346170
Staphylococcus epidermidis	HD26-1	No	Yes	No	33544760
Staphylococcus epidermidis	HD27-3	No	Yes	No	33544760
Staphylococcus epidermidis	RP62A	No	Yes	No	15774886
Staphylococcus equorum	KS1039	No	Yes	No	26718562
Staphylococcus felis	ATCC49168	Yes	No	Yes	29773625
Staphylococcus felis	FDAARGOS_1014	Yes	No	Yes	31346170
Staphylococcus fleurettii	ssch2	Yes	No	No	N/A
Staphylococcus haemolyticus	GDY8P80P	Yes	No	Yes	34069037
Staphylococcus hyicus	ATCC11249	Yes	No	No	25700402
Staphylococcus hyicus	NCTC10350	Yes	No	No	31023808
Staphylococcus lugdunensis	C_33	Yes	No	No	28444231
Staphylococcus lugdunensis	NCTC7990	Yes	No	Yes	N/A
Staphylococcus lugdunensis	SL117	No	Yes	No	31736914
Staphylococcus lugdunensis	SL122	No	Yes	No	31736914
Staphylococcus lugdunensis	SL13	No	Yes	Yes	31736914
Staphylococcus lugdunensis	SL29	No	Yes	No	31736914
Staphylococcus lugdunensis	VISLISI_27	No	Yes	Yes	28444231
Staphylococcus lugdunensis	VISLISI_33	No	Yes	Yes	28444231
Staphylococcus lugdunensis	VISLISI_37	No	Yes	No	28444231
Staphylococcus lugdunensis	HKU09-01	No	Yes	Yes	20047907
Staphylococcus lugdunensis	N920143	No	Yes	No	21682763
Staphylococcus lutrae	ATCC 700373	Yes	No	No	28642379

Staphylococcus pasteuri	SP1	Yes	No	Yes	N/A
Staphylococcus piscifermentans	NCTC13836	Yes	No	No	1390108
Staphylococcus pseudintermedius	HSP080	Yes	No	Yes	34796561
Staphylococcus pseudintermedius	HSP125	Yes	No	Yes	34796561
Staphylococcus pseudintermedius	HSP204	Yes	No	No	34796561
Staphylococcus pseudintermedius	HSP210	Yes	No	No	34796561
Staphylococcus pseudintermedius	HSP211	Yes	No	No	34796561
Staphylococcus pseudintermedius	HSP212	Yes	No	No	34796561
Staphylococcus pseudintermedius	HSP213	Yes	No	No	34796561
Staphylococcus pseudintermedius	HSP216	Yes	Yes	Yes	34796561
Staphylococcus pseudintermedius	HSP240	Yes	Yes	No	34796561
Staphylococcus pseudintermedius	HSP252	Yes	No	No	34796561
Staphylococcus pseudintermedius	HSP274	Yes	No	Yes	34796561
Staphylococcus pseudintermedius	HSP276	Yes	No	Yes	34796561
Staphylococcus pseudintermedius	HSP277	Yes	No	No	34796561
Staphylococcus pseudintermedius	HSP278	Yes	No	Yes	34796561
Staphylococcus pseudintermedius	HSP279	Yes	No	No	34796561
Staphylococcus pseudintermedius	HSP280	Yes	No	No	34796561
Staphylococcus pseudintermedius	HSP282	Yes	No	Yes	34796561
Staphylococcus pseudintermedius	HSP284	Yes	No	Yes	34796561
Staphylococcus pseudintermedius	63228	No	Yes	No	27795289
Staphylococcus pseudintermedius	157588	Yes	Yes	No	N/A
Staphylococcus pseudintermedius	51_92	Yes	No	No	31296692
Staphylococcus pseudintermedius	53_60	Yes	No	No	31296692
Staphylococcus pseudintermedius	53_88	Yes	No	Yes	31296692
Staphylococcus pseudintermedius	DG099	Yes	No	No	34796561
Staphylococcus pseudintermedius	DSP030	No	Yes	Yes	34796561
Staphylococcus pseudintermedius	DSP034	Yes	No	No	34796561
Staphylococcus pseudintermedius	HSP079	Yes	No	Yes	34796561
Staphylococcus pseudintermedius	HSP118	Yes	No	No	34796561
Staphylococcus pseudintermedius	HSP132	Yes	No	No	34796561
Staphylococcus pseudintermedius	HSP134	Yes	No	No	34796561
Staphylococcus pseudintermedius	HSP135	Yes	No	No	34796561
Staphylococcus pseudintermedius	HSP136	Yes	No	No	34796561
Staphylococcus pseudintermedius	HSP137	Yes	No	No	34796561
Staphylococcus pseudintermedius	HSP138	Yes	No	No	34796561
Staphylococcus pseudintermedius	K18PSP147	No	Yes	No	N/A
Staphylococcus pseudintermedius	MAD401	No	Yes	No	29650582
Staphylococcus pseudintermedius	MAD627	Yes	No	No	29650582
Staphylococcus pseudintermedius	ME4692	Yes	No	Yes	29650582
Staphylococcus pseudintermedius	SP79	Yes	No	No	N/A
Staphylococcus pseudintermedius	SP_11304-2A	Yes	Yes	No	34165335
Staphylococcus pseudintermedius	SP_11306-4A	Yes	No	No	34165335
Staphylococcus pseudintermedius	ED99	Yes	No	Yes	21398539
Staphylococcus ratti	CCM 9025	Yes	No	No	35055999
Staphylococcus schleiferi	1360-13	Yes	No	No	26358596
Staphylococcus schleiferi	2142-05	Yes	No	No	26358596
Staphylococcus schleiferi	2317-03	Yes	No	Yes	26358596
Staphylococcus schleiferi	5909-02	Yes	No	Yes	26358596
Staphylococcus schleiferi	NCTC12218	Yes	No	No	31023808
Staphylococcus schleiferi	TSCC54	Yes	Yes	No	26514766
Staphylococcus simulans	FDAARGOS_124	Yes	No	Yes	31346170
Staphylococcus simulans	FDAARGOS_383	Yes	No	Yes	31346170

Staphylococcus simulans	MR1	Yes	No	No	28743956
Staphylococcus simulans	MR2	Yes	No	No	28743956
Staphylococcus simulans	MR3	Yes	No	No	28743956
Staphylococcus simulans	MR4	Yes	No	No	28743956
Staphylococcus simulans	NCTC11046	Yes	No	No	31023808
Staphylococcus simulans	NCTC7944	Yes	No	Yes	N/A
Staphylococcus spp.	17KM0847	Yes	No	No	N/A
Staphylococcus spp.	HL28	Yes	No	Yes	N/A
Staphylococcus spp.	M0911	Yes	No	Yes	N/A
Staphylococcus spp.	MI 10-1553	Yes	Yes	Yes	32672529
Staphylococcus warneri	22.1	Yes	No	Yes	N/A
Staphylococcus warneri	NCTC4133	Yes	No	Yes	N/A
Staphylococcus warneri	WS479	Yes	No	Yes	34394031

Table S2. Bacterial strains used in this study. Related to STAR Methods.

Species	Strain	Genotype	Origin and Construction Notes
S. aureus	RN4220	NCTC8325 cured of all three prophages and inactivated restriction subunit (*hsdR*) of R-M system to facilitate genetic manipulation	Kreiswerth et al., *Nature* (1983)
S. aureus	RN10616	RN4220::Φ80α	Ubeda et al., *Mol Microbiol* (2009)
S. aureus	RN10628	RN4220::Φ80α::SaPI1 *tst*::*tetM*	Ubeda et al., *Mol Microbiol* (2009)
S. aureus	OS2	RN4220 *spa*::*ermC*	Schneewind et al., *Cell* (1992)
S. aureus	ST16	RN4220::Φ80α Δ*terS*::SaPI1 *tst*::*tetM*	Ubeda et al., *Mol Microbiol* (2009)
S. aureus	ST24	RN4220::Φ80α Δ*terS*	Ubeda et al., *Mol Microbiol* (2009)
S. aureus	ST126	RN4220::Φ80α::SaPI1 *tst*::*tetM* Δ*cpmAB*	Damle et al., *Virology* (2012)
S. aureus	TB4	Newman cured of all four prophages (ΦNM1-4)	Bae et al., *Mol Microbiol* (2006)
S. aureus	DVB3	TB4::ΦNM1:: SaPI1 *tst*::*tetM*	This study; lysogenization of TB4 by ΦNM1, followed by transduction by purified SaPI1 *tst*::*tetM* particles (generated from mitomycin C-induction of ST16)
S. aureus	DVB6	TB4::Φ80α	This study; lysogenization of TB4 by Φ80α (generated from mitomycin C-induction of RN10616)
S. aureus	DVB7	TB4::SaPI1 *tst*::*tetM*	This study; transduction of TB4 by purified SaPI1 *tst*::*tetM* particles (generated from mitomycin C-induction of ST16)
S. aureus	DVB8	TB4::Φ80α::SaPI1 *tst*::*tetM*	This study; transduction of DVB6 by purified SaPI1 *tst*::*tetM* particles (generated from mitomycin C-induction of ST16)
S. aureus	DVB11	RN4220::Φ80α Δ*terS*::SaPI1 *tst*::*tetM* Δ*cpmAB*	This study; transduction of ST24 by SaPI1 *tst*::*tetM* Δ*cpmAB* particles (generated from mitomycin C-induction of ST126)
S. aureus	DVB16	TB4::SaPI1 *tst*::*tetM* Δ*cpmAB*	This study; transduction of TB4 by purified SaPI1

			tst::*tetM* Δ*cpmAB* particles (generated from mitomycin C-induction of DVB11)
S. aureus	DVB21	TB4::Φ80α::SaPI1 *tst*::*tetM* Δ*cpmAB*	This study; transduction of DVB6 by purified SaPI1 *tst*::*tetM* Δ*cpmAB* particles (generated from mitomycin C-induction of DVB11)

Table S3. Phages used in this study. Related to STAR Methods.

Phage	Host	Genotype	Origin
Φ80α-vir	*S. aureus*	Wild type	Banh et al., *Nature* (2023); strictly lytic mutant of Φ80α isolated from type III CRISPR-Cas targeting of *cI* repressor gene
Φ80α-vir SaPI1-escaper #1	*S. aureus*	*sri* E24* (G70>T)	This study; isolated as escaper plaque on DVB7
Φ80α-vir(21^{seed})	*S. aureus*	*gp21* L157 (silent) (T469>C)	This study; isolated as escaper plaque on TB4/pDVB52
Φ80α-vir(21^{PAM})	*S. aureus*	*gp21* M158I (G474>T)	This study; isolated as escaper plaque on TB4/pDVB52

Table S4. Plasmids used in this study. Related to STAR Methods.

Plasmid	Description	Source	Construction Notes
pC194	Medium-copy plasmid in staphylococci and shuttle vector encoding chloramphenicol resistance	Horinouchi et al., *J Bacteriol* (1982)	Natural isolate
pDVB08	*S. epidermidis* RP62a type III-A CRISPR-Cas system (native promoter) with programmed spacer targeting Φ80α *cI* gene and native promoter	Banh et al., *Nature* (2023)	Ligation of BsaI-digested vector pGG78 and annealed oligos oDVB16/oDVB17
pDVB47; designated pCRISPR-II (*spcNT*)	*S. aureus* M06/0171 type II-A CRISPR-Cas system (native promoter) with non-targeting spacer and BsaI cloning site	This study	Gibson Assembly: oDVB96+128, oDVB97+129, oDVB126+127 (all using pDB236 template)
pDVB52; designated pCRISPR-II (*spc21*)	*S. aureus* M06/0171 type II-A CRISPR-Cas system (native promoter) with programmed spacer targeting Φ80α *gp21*	This study	Ligation of BsaI-digested vector pDVB47 and annealed oligos oDVB148/oDVB149
pDVB55; designated pCRISPR-III (*spc14*)	*S. epidermidis* RP62a type III-A CRISPR-Cas system (native promoter) with programmed spacer targeting Φ80α *gp14*	This study	Ligation of BsaI-digested vector pGG78 and annealed oligos oDVB154/oDVB155
pDVB59; designated pCRISPR-III (*spcNT, dcsm6*)	*S. epidermidis* RP62a type III-A CRISPR-Cas system d*csm6* (native promoter) with non-targeting spacer and BsaI cloning site	This study	Gibson Assembly: oDVB162+163, oDVB164+165 (both using pGG78 template)
pDVB61; designated pCRISPR-III (*spc14, dcsm6*)	*S. epidermidis* RP62a type III-A CRISPR-Cas system d*csm6* (native promoter) with programmed spacer targeting Φ80α *gp14*	This study	Ligation of BsaI-digested vector pDVB59 and annealed oligos oDVB154/oDVB155
pDVB96; designated pCRISPR-II (*spc21, spc05*)	*S. aureus* M06/0171 type II-A CRISPR-Cas system (native promoter) with two programmed spacers targeting Φ80α gp21 *and gp05*	This study	Phage-resistant (CRISPR adapted) clone harboring pDVB52 isolated during infection by Φ80α-vir$^{gp21\ \mathrm{PAM}}$
pDVB99; designated pCRISPR-II	*S. aureus* M06/0171 type II-A CRISPR-Cas system (native promoter) with a single repeat	This study	Gibson Assembly: oDVB534+535 (pDVB47 template)
pDVB119; designated pCRISPR-II (Δ*cas1*)	*S. aureus* M06/0171 type II-A CRISPR-Cas system Δ*cas1* (native promoter) with a single repeat	This study	Gibson Assembly: oDVB577+578 (pDVB99 template)
pGG-BsaI-R; designated pCRISPR-III (*spcNT*)	*S. epidermidis* RP62a type III-A CRISPR-Cas system (native promoter) with non-	Jiang et al., *Cell* (2016)	"Round-the-horn" PCR followed by blunt-end ligation, oGG281+oGG282 (pGG-BsaI-R template)

	targeting spacer and BsaI cloning site		
pWJ191; designated pCRISPR-III (*spc47*)	*S. epidermidis* RP62a type III-A CRISPR-Cas system (native promoter) with programmed spacer targeting Φ80α *gp47*	Jiang et al., *Cell* (2016)	Ligation of BsaI-digested vector pGG-BsaI-R and annealed oligos oGG250/oGG251
pWJ241; designated pCRISPR-III (*spc47, dcsm6*)	*S. epidermidis* RP62a type III-A CRISPR-Cas system d*csm6* (native promoter) with programmed spacer targeting Φ80α *gp47*	Jiang et al., *Cell* (2016)	Ligation of BsaI-digested vector pGG97 and annealed oligos oGG250/oGG251

Table S5. Oligonucleotide primers used in this study. Related to STAR Methods.

Primer	Sequence
oDVB16	GAACATTGCTCGTTTGCATAGTTAAGCACATTTTG
oDVB17	CGATCAAAATGTGCTTAACTATGCAAACGAGCAAT
oDVB96	AGCATTTTCAGGTATAGGTGTTTTGGGAAACAATTTCCCCGAACCATTATATTTCTCTAC
oDVB97	TTTCCCAAAACACCTATACCTGAAAATGCTTTTTCTCTTTCTATTATTCCATGGACTTCA
oDVB126	TGAAAAAATTAAGCTGCACGATATGCAAGAAGGGAAATGTTTATACTCGTTAGAAGCAAT
oDVB127	GAGACCTTTGAGCTTCCGAGACTGGTCTCAGTCTACCTCATACCTAAAATTACAGAGTAC TAAAACCTCTAGCATCATTATAAATCCTCA
oDVB128	TGAGACCAGTCTCGGAAGCTCAAAGGTCTCGTTTTAGTACTCTGTAATTTTAGGTATGAG GTAGACAGCAAAATCACTGATAGGGAGAGG
oDVB129	TCTTGCATATCGTGCAGCTTAATTTTTTCAATTAAATATTTAGCATTTTCTTTACCTGTC
oDVB148	AGACACGGTTGCTTTTATGCACATACCAATGTTGG
oDVB149	AAAACCAACATTGGTATGTGCATAAAAGCAACCGT
oDVB154	GAACTAACATGAATATTTTTGCGTATGATTTCGCCTATTG
oDVB155	CGATCAATAGGCGAAATCATACGCAAAAATATTCATGTTA
oDVB162	TGGTTTAGCAAATTCCATAGCCGCTAATTTAGATACATTAAATTTGGATAAAAATAAAAA
oDVB163	TTTATTAATGAATTTTCCTGCTGTAATAATGGGTAGAAGGTAATTACTATTATTATTGAT
oDVB164	ATTATTACAGCAGGAAAATTCATTAATAAAGGTAATTCAATATATTTACCGCTATCTTTA
oDVB165	AAATTAGCGGCTATGGAATTTGCTAAACCATTTATATCGTTTACTGCATTGACTTCTTTT
oDVB225	AGGATTTATAATGATGCTAGAG
oDVB420	CTGCTTTCTTCATTAGAATCAA
oDVB534	GTTTTAGTACTCTGTAATTTTAGGTATGAGGTAGACAGCAAAATCACTGATAGGGAGAGG
oDVB535	GTCTACCTCATACCTAAAATTACAGAGTACTAAAACCTCTAGCATCATTATAAATCCTCA
oDVB577	ATAACCTCTAGGCTTTTAAGCAATATTCATTTTATAAATCATGAGTTATAAGTTTATGAG
oDVB578	ATGAATATTGCTTAAAAGCCTAGAGGTTATAGTAGTTGTTAAATC
oDVB658	ATTTGTCAAAAAAAGTGACATATCA
oDVB659	CAAAATCTACAATTTTTTAGAGTGT
oGG250	GAACATTCGTCATCTTCAAGTAATGCCTCTAAATCAATA
oGG251	GATCTATTGATTTAGAGGCATTACTTGAAGATGACGAAT
oGG281	GTATCGATCGAGACCTTTGAGCTTCCGAGAC
oGG282	CCACCCCGAAGAAAAGGGGACGAGAACTAAATCTAACAACACTCTAAAAAATTG
oGG338	CTTGTTCAAATTACTTTCGTCTG
oGG339	CTTAAAATTATTCGTTGATGCAGG
oGG340	CCTTGAACTTCCTTGATAATCTG
oGG341	ATTCTCACGTTACTGAACAGATG
